# Quercetin as a therapeutic agent activate the Nrf2/Keap1 pathway to alleviate lung ischemia-reperfusion injury

**DOI:** 10.1038/s41598-024-73075-7

**Published:** 2024-10-04

**Authors:** Mohammad Yousefi Zardak, Fatemeh Keshavarz, Ali Mahyaei, Morteza Gholami, Fatemeh Sadat Moosavi, Elham Abbasloo, Farzaneh Abdollahi, Maryam Hossein Rezaei, Elham Madadizadeh, Nasrin Soltani, Fatemeh Bejeshk, Niyan Salehi, Fahimeh Rostamabadi, Fatemeh Bagheri, Mahla Jafaraghae, Mahdiyeh Ranjbar Zeydabadi, Meraj Baghgoli, Gholamreza Sepehri, Mohammad Abbas Bejeshk

**Affiliations:** 1 Physiology Research Center, Institute of Neuropharmacology, Kerman University of Medical Sciences, Kerman, Iran; 2 Department of Physiology and Pharmacology, Afzalipour Medical Faculty, Kerman University of Medical Sciences, Kerman, Iran; 3 Pulmonary Research Center, Institute of Basic and Clinical Physiology Sciences, Kerman University of Medical Sciences, Kerman, Iran; 4 Endocrinology and Metabolism Research Center, Institute of Basic and Clinical Physiology Sciences, Kerman University of Medical Sciences, Kerman, Iran; 5 Department of Exercise Physiology, Faculty of Physical Education, Shahid Bahonar University, Kerman, Iran; 6 Department of Biology, Faculty of Sciences, Shahid Bahonar University of Kerman, Kerman, Iran; 7 Noncommunicable Diseases Research center, Bam University of Medical Sciences, Bam, Kerman Iran; 8 Legal Medicine Research Center, Legal Medicine Organization, Kerman, Iran; 9 Faculty of Mathematics and Computer Science, Shahid Bahonar University, Kerman, Iran; 10 Neuroscience Research Center, Kerman University of Medical Sciences, Kerman, Iran

**Keywords:** Lung ischemia-reperfusion injury, Oxidative stress, Inflammation, Nrf2/Keap1 pathway, Quercetin, Physiology, Apoptosis

## Abstract

Lung ischemia-reperfusion injury (LIRI) causes oxidative stress, inflammation, and immune system activation. The Nrf2/Keap1/HO-1 pathway is important in cellular defense against these effects. Quercetin, a flavonoid with antioxidant, anti-inflammatory, and anti-cancer properties, has been investigated. Our aim in this study was to investigate the effect of quercetin on preventing lung ischemia-reperfusion injury and the role of the Nrf2/Keap1/HO-1 pathway. Sixty-four male Wistar rats were divided into four distinct groups(*n* = 16). Sham, lung ischemia-reperfusion (LIR), Saline + LIR, Quercetin + LIR (30 mg/kg i.p for a week before LIR). LIR groups were subjected to 60 min of ischemia (left pulmonary artery, vein, and bronchus) and 120 min of reperfusion. Our assessment encompassed a comprehensive analysis of various factors, including the evaluation of expression Nrf2, Keap1, and Heme Oxygenase-1 (HO-1) levels and NF-κB protein. Furthermore, we examined markers related to inflammation (interleukin-1β and tumor necrosis factor alpha), oxidative stress (malondialdehyde, total oxidant status, superoxide dismutase, glutathione peroxidase, total antioxidant capacity), lung edema (Wet/dry lung weight ratio and total protein concentration), apoptosis (Bax and Bcl2 protein), and histopathological alterations (intra-alveolar edema, alveolar hemorrhage, and neutrophil infiltration). Our results show that ischemia-reperfusion results in heightened inflammation, oxidative stress, apoptosis, lung edema, and histopathological damage. Quercetin showed preventive effects by reducing these markers, acting through modulation of the Nrf2/Keap1 pathway and inhibiting the NF-κB pathway. This anti-inflammatory effect, complementary to the antioxidant effects of quercetin, provides a multifaceted approach to cell protection that is important for developing therapeutic strategies against ischemia-reperfusion injury and could be helpful in preventive strategies against ischemia-reperfusion.

## Introduction

Lung ischemia-reperfusion Injury (LIRI) can occur following lung transplantation and various clinical conditions such as trauma, arterial sclerosis, cerebral infarctions, myocardial infarctions, embolism, and pulmonary thrombosis^1^. It commonly complicates the clinical course in certain conditions like heart-lung bypass surgeries and complex grafting procedures, altering prognosis and outcomes^2,3^. Annually, over 4600 lung transplants are performed worldwide, yet one-third of all lung transplant surgeries result in primary graft dysfunction (PGD) within 72 h, contributing to 50% of first-year post-transplant mortality4. Moreover, even those who survive PGD experience reduced lung graft function and an increased risk of developing obstructive bronchiolitis syndrome (OBS)^4^. During the period of lung ischemia, the lack of oxygen and nutrients creates conditions that, upon reperfusion, lead to oxidative stress, calcium overload, endoplasmic reticulum stress, coagulation disorders, autophagy, apoptosis, activation of innate immunity, production of inflammatory cytokines, and leukocyte recruitment^2,5^.

The Nuclear factor-erythroid factor 2-related factor 2/ Kelch-like ECH-associated protein 1/ Heme oxygenase 1 (Nrf2/Keap1/HO-1) pathway is a crucial cellular mechanism that safeguards cells against oxidative stress, apoptosis, and inflammation^6,7^. Nrf2 is a regulatory protein responsible for gene expression in cellular protection. It is bound to Keap1 in the cytoplasm, and under stress conditions, Nrf2 dissociates from Keap1, translocates into the nucleus, and induces the expression of defensive genes such as HO-1^6,7^. HO-1 is a protective enzyme that catalyzes heme into carbon monoxide, free iron, and biliverdin, imparting anti-apoptotic, anti-inflammatory, and antioxidant effects. Activation of this pathway might shield cells against oxidative stress, apoptosis, and inflammation resulting from ischemia and reperfusion injury^2–5,7^.

Quercetin is a type of flavonoid found in apples, tea, onions, nuts, berries, cauliflower, cabbage, and many other foods. In studies conducted by others on the properties of quercetin, this compound has numerous benefits for promoting health. These benefits include improving cardiovascular health, addressing eye diseases, allergies, arthritis, reducing the risk of cancer, and many other conditions^8^. This flavonoid is a potent tissue-rejuvenating agent, protecting the body against oxidative stress. Additionally, other research suggests that quercetin acts as an antioxidant, anti-inflammatory, prooxidant, induces apoptosis in cancer cells, and prevents tumor proliferation. Other properties of quercetin include its ability to modulate genes related to the cell cycle, signal transduction, xenobiotic metabolism, antiviral, anti-inflammatory, antibacterial effects, and muscle relaxation^9–12^.

Recently, a definitive treatment for LIRI has yet to be introduced, and the only practical approaches are symptomatic and supportive treatments. Considering the properties of quercetin and its protective effects on the Nrf2/Keap1 pathway in cells against oxidative stress, apoptosis, and inflammation prompted us to investigate the potential of intraperitoneal injection of quercetin in controlling the consequences of ischemia and reperfusion-induced injuries by affecting the Nrf2/Keap1/HO-1 pathway.

## Materials and methods

### Animal selection

This study used adult male Wistar rats weighing between 190 and 210 g. The animals were obtained from the Kerman University of Medical Sciences’ animal farm and housed in the Faculty of Medicine’s animal house under a 12-hour light/dark cycle, an ambient temperature of 23 ± 2 °C, and unrestricted access to water and food. The experiments adhered to ethical guidelines for animal investigation, with the protocol approved by the Ethics Committee of Kerman University of Medical Sciences. We implemented established principles to ensure the animals’ welfare and took all necessary measures to minimize their discomfort and suffering.

### Study design

This study was conducted using a pre-treatment design. The drug quercetin (Sigma-Aldrich, Germany) at a dose of 30 mg/kg^13–15^ was administered through intraperitoneal injection for seven consecutive days. The animals were divided into four main groups, each with 16 rats.

Group Sham: In this group, surgical procedures, including tracheostomy and exposing the lung hilum, were performed, but the left pulmonary artery, vein, and bronchus were not obstructed. Group Lung Ischemia-Reperfusion (LIR): Similar to the Sham group, the left pulmonary artery, vein, and bronchus were obstructed for 60 min, followed by a reperfusion period of 120 min. Group Saline + LIR: Animals in this group received consecutive doses of 5% ethanol for seven days and then underwent ischemia-reperfusion. Group Quercetin + LIR: Animals in this group received quercetin at a dose of 30 mg/kg^13–15^ for seven consecutive days and then underwent ischemia-reperfusion.

### Anesthesia, pain management, and euthanize in animals

The animals were kept anesthetized using a cocktail of ketamine (80 mg/kg) and xylazine (10 mg/kg) for induction, followed by halothane (2%) and nitrous oxide (N_2_O) for maintenance during surgery. At the end of the reperfusion stage, our animals were euthanized by increasing the anesthetic dose, we deepened the anesthesia until cardiac function ceased, ensuring a painless death.

### Tracheostomy

After shaving the neck and chest, a midline incision was made from the jugular notch to the hyoid bone using a size 22 scalpel. The inferior thyroid artery, recurrent laryngeal nerve, and vagus nerve were separated. Then, a half-circumference incision was made between the cartilages, and a tracheal tube was inserted and secured with sutures. The ventilation machine was connected to the tracheal tube, and the animal’s breathing was controlled at 60–70 breaths per minute with a tidal volume of 6 to 8 mg/kg^16^.

### Induction of ischemia and reperfusion

Animals were positioned in the right lateral decubitus position, and the space between the fifth rib was incised to expose the lung. The left pulmonary hilum was dissected, and a non-invasive microvascular clamp was used to block it. Before the occlusion, 100 international units of heparin were injected intravenously. After 60 min, the clamp was removed, and 120 min of reperfusion was initiated. To maintain body fluids, 0.5 ml of normal saline was injected into the animals every hour^17^.

### Collecting bronchoalveolar iavage fluid (BALF)

At the end of the study, after removing the heart block and ligating the right lung hilum, a chip cannula attached to a syringe was inserted into the trachea. The left lung was lavaged with 5 ml of phosphate-buffered saline (PBS) followed by lavage fluid^18^.

### Measurement of total protein content in BALF

The total protein content in the bronchoalveolar lavage fluid (BALF) was assessed as another indicator of lung edema. BALF protein concentration was measured using a bicinchoninic acid (BCA) protein assay. This measurement was carried out using a commercially available kit provided by the manufacturer, Navand Salamat Company. The assay was conducted according to the manufacturer’s instructions, as outlined in reference^19^.

### Measurement of wet/dry lung weight ratio

The initial step in assessing lung edema was determining the wet/dry lung weight ratio^19^. This ratio is indicative of the amount of fluid accumulation in the lungs. The lungs were first weighed while still wet. Subsequently, the lung tissue was subjected to drying to remove any moisture. This was accomplished by incubating the lung at 60 °C for 48 h. This process ensures that the lung tissue is completely devoid of moisture^19^.

### Inflammatory assessment

The enzyme-linked immunosorbent assay (ELISA) technique was utilized to measure the cytokines TNF-α (Karmania Pars Gen, Kerman, Iran, KPG-RTNF-α) and IL-1β (Karmania Pars Gen, Kerman, Iran, KPG-RIL-1β) in lung tissue. In summary, 50–100 milligrams of lung tissue were homogenized in PBS using ultrasonication, followed by centrifugation at 2000 rpm for 20 min. The resulting supernatant was collected, and the levels of the mentioned cytokines were measured according to the manufacturer’s kit instructions^19^.

### Evaluation of oxidative stress

Superoxide dismutase (SOD) (Navand Salamat Co, Iran, NS-15033) glutathione peroxidase (GPX) (Navand Salamat Co, Iran, NS-15083), malondialdehyde (MDA) (Behboud Tahghigh Co, Kerman, Iran NS-BTK1991), total oxidant status (TOS) (Navand Salamat Co, Iran, NS-15017) and total antioxidant capacity (TAC) (Behboud Tahghigh Co, Kerman, Iran NS-BTK1992), were measured by their specific kits according to the manufacturer’s instructions^19^.

### Evaluation of protein expression with western blotting

To investigate the expression of proteins Bax, Bcl2, NF-kb, Nrf2, Ho-1(Abcam, USA, ab32503, ab237892, ab239882, ab313825, ab305290) and Keap1(Bio techne, USA, MAB3024), the Western blotting technique was employed. Briefly, the Bradford method was used to measure the total protein content (Supplementary information). Forty micrograms of each sample were loaded onto a 12% SDS-PAGE gel and subjected to electrophoresis. The separated proteins were then transferred to a PVDF membrane. Subsequently, the membrane was incubated with the appropriate primary (1:500) and secondary (1:10000) antibodies. Finally, the antibody-antigen complex was visualized using the Enhanced Chemiluminescence (ECL) system and captured by a gel documentation apparatus. The band densities were quantified using Image J software version 1.8.0(https://imagej.en.download.it/download). In the final step, the PVDF membrane was subjected to stripping solution at 55 degrees Celsius for half an hour, followed by blocking again with TBST for beta-actin^20^.

### Histopathological evaluation

A portion of the left lung was fixed in 10% formaldehyde, and four pathological sections were prepared. Hematoxylin and eosin staining were employed to examine intra-alveolar edema, alveolar hemorrhage, interstitial congestion, and neutrophil infiltration. Subsequently, a blinded assessment of lung tissue histopathological changes was conducted by a pathologist using a 5-point scoring system. The scores were as follows: 0: no change, 1: 0 to 25% change, 2: 25 to 50% change, 3: 50 to 75% change, 4: 75 to 100% change^21^.

### Statistical analysis

Graph Pad Prism v.6 software was used for statistical analysis. The Shapiro-Wilk test assessed data distribution. A one-way ANOVA with Tukey’s post hoc test was used for normally distributed quantitative data, while the Kruskal-Wallis test analyzed non-parametric data. A significance level of *P* < 0.05 was deemed statistically significant.

## Results

### The effects of quercetin on lung edema

**Fig. 1:**
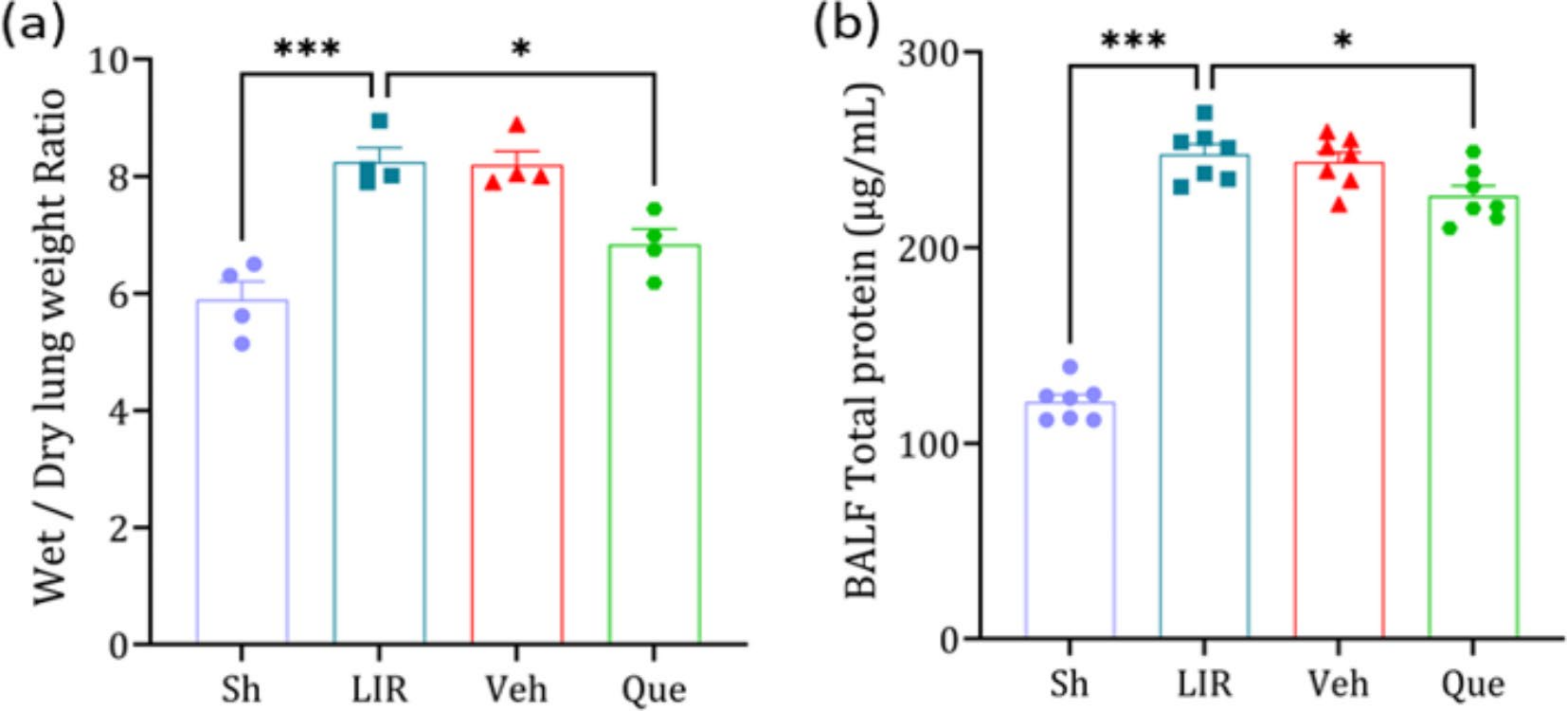
The effects of quercetin on wet-to-dry weight ratio(*n* = 6) and total protein content)*n* = 4). The data was presented as means ± SEM.* BALF* bronchoalveolar lavage fluid,* Sh* sham group,* LIR* lung ischemia-reperfusion group,* Veh* vehicle group,* Que* quercetin group. * *P* < 0.05, *** *P* < 0.001.

The lung wet-to-dry weight ratio and total protein content in BALF significantly increased in the lung ischemia-reperfusion (LIR) group compared to the sham group. However, one week of quercetin pretreatment prevented this increase in the quercetin group relative to the LIR group (Fig. 1, panels a,b).

### The effects of quercetin on mediators of inflammation and oxidative stress factors

**Fig. 2:**
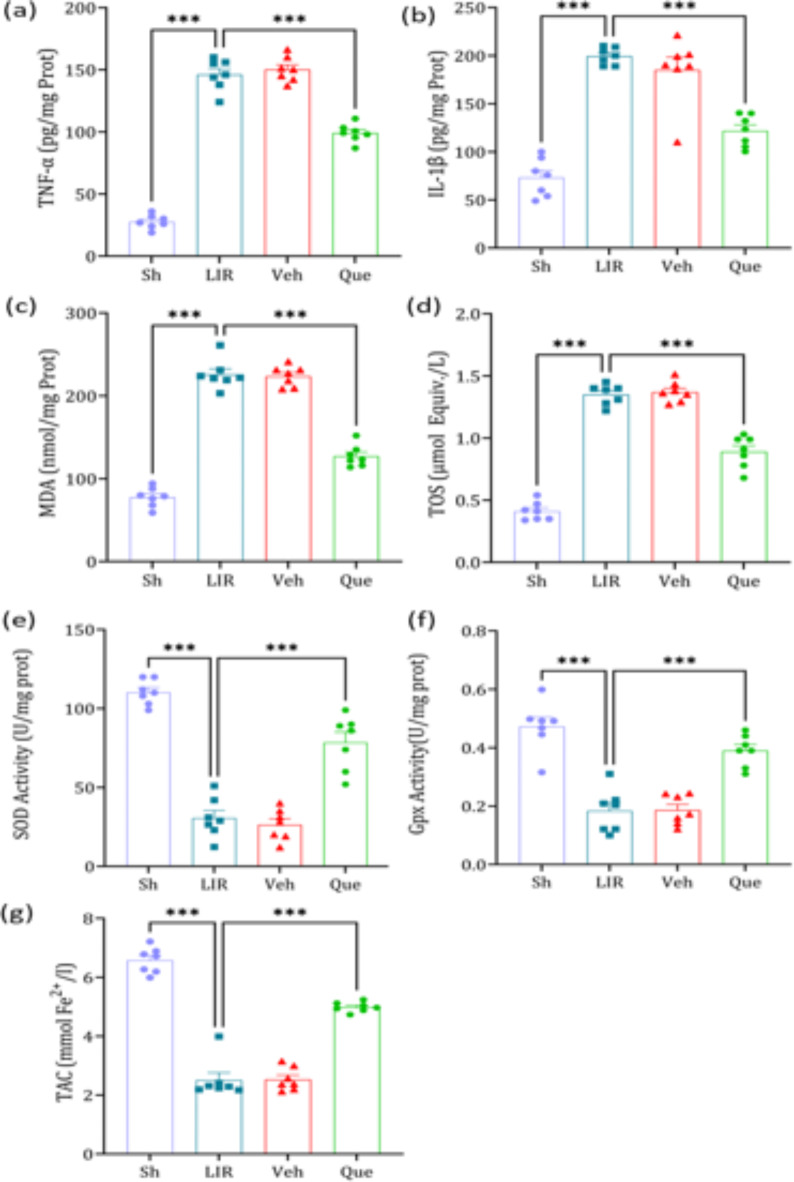
The effects of quercetin on oxidative stress and inflammation factors in lung tissue. The data was presented as means ± SEM, (*n* = 6).* Sh* sham group,* LIR* lung ischemia-reperfusion group,* Veh* vehicle group,* Que* quercetin group. *** *P* < 0.001.

The levels of inflammatory markers, IL-1β and TNF-α, were significantly increased in these markers in the LIR group compared to the sham group. In addition, pretreatment with quercetin resulted in a significant decrease in IL-1β and TNF-α levels compared to the LIR group (Fig. 2. Panels a and b).

The tissue levels of oxidative markers MDA and TOS were significantly increased in the LIR group compared to the sham group. In addition, the antioxidant markers, SOD, GPX, and TAC, were significantly decreased in the LIR group compared to the sham group. Evaluation of oxidative agents MDA and TOS showed a significant decrease in these agents in the quercetin group compared to the LIR group. In addition, a significant increase in the levels of antioxidant factors SOD, TAC, and GPX was observed in the quercetin injection group compared to the LIR group (Fig. 2. Panels c–g).

### The effects of quercetin on the expression of bax, Bcl_2_, NF-κB, Nrf2, HO-1, and Keap1 proteins

The expression levels of apoptosis-related proteins Bax and Bcl2 showed a significant increase in the LIR group compared to the Sham group. This study demonstrated novel findings that LIR also influences the expression levels of tissue regulators; indeed, factors such as NF-κB and Keap1 exhibited a significant increase in the LIR group compared to the Sham group. In addition, the expression of Nrf2 and HO-1 decreased in the LIR compared to the sham group.

Investigations within the Quercetin-pretreated group revealed significant alterations in Bax, Bcl2, NF-κB, Nrf2, HO-1, and Keap1 protein expression levels. Compared to the LIR group, the protein levels of the pro-apoptotic protein Bax were notably reduced, whereas the expression of the apoptosis-inhibiting protein Bcl2 exhibited a substantial increase.

**Fig. 3:**
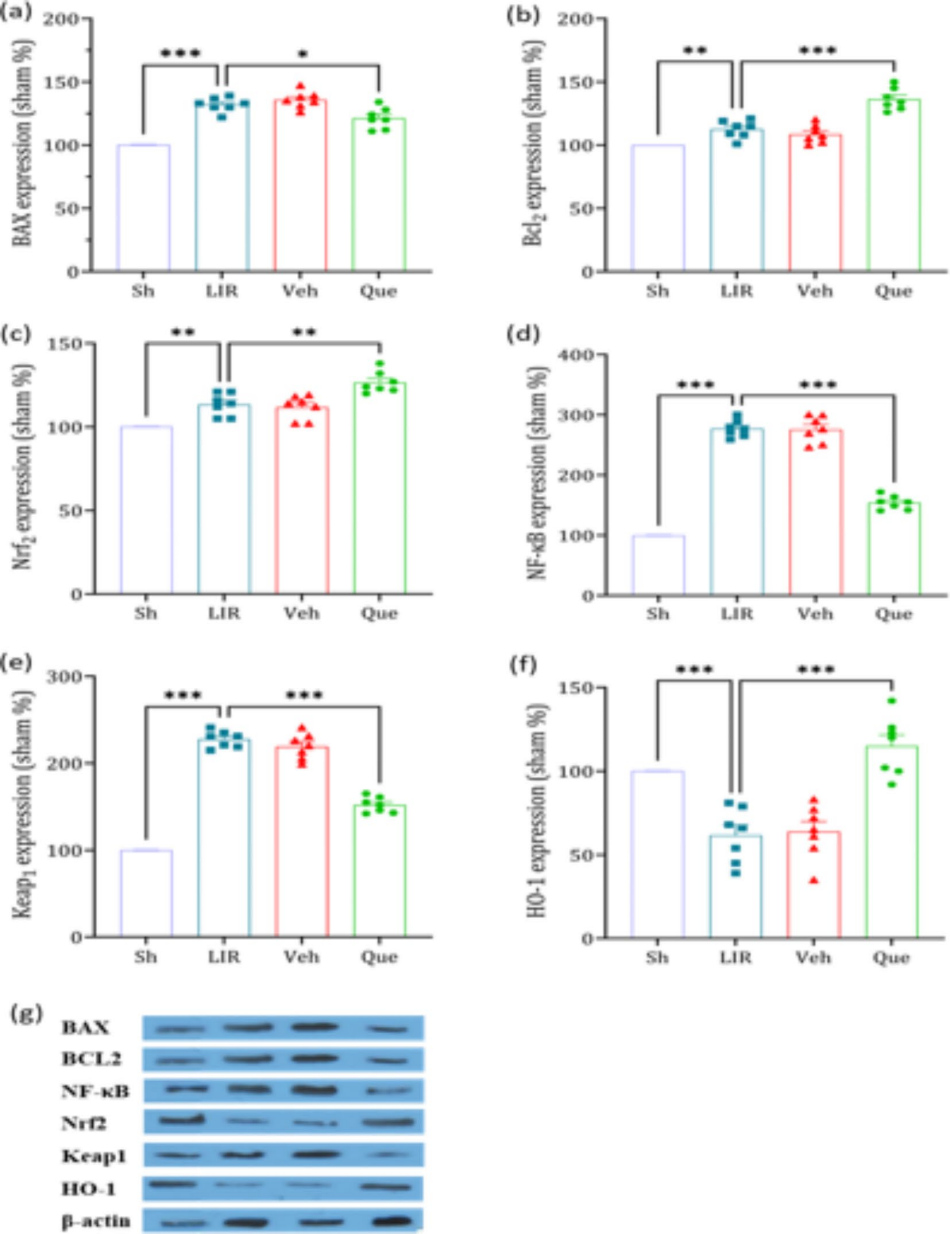
The effect of quercetin on the expression of Bax, Bcl2, NF-κB, Nrf2, HO-1 and Keap1 proteins in lung tissue. The data was presented as means ± SEM, (*n* = 6). * Sh* sham group,* LIR* lung ischemia-reperfusion group,* Veh* vehicle group,* Que* quercetin group. * *P* < 0.05, ** *P* < 0.01, *** *P* < 0.001.

Furthermore, Quercetin influenced the tissue levels of NF-κB, Nrf2, HO-1, and Keap1. Following its administration to rats, the results showed a significant reduction in the levels of NF-κB and Keap1 factors. Conversely, the levels of Nrf2 and HO-1 increased contrary to the aforementioned factors (Fig. 3, panels a–f).

### The effects of quercetin on lung histopathology

**Fig. 4:**
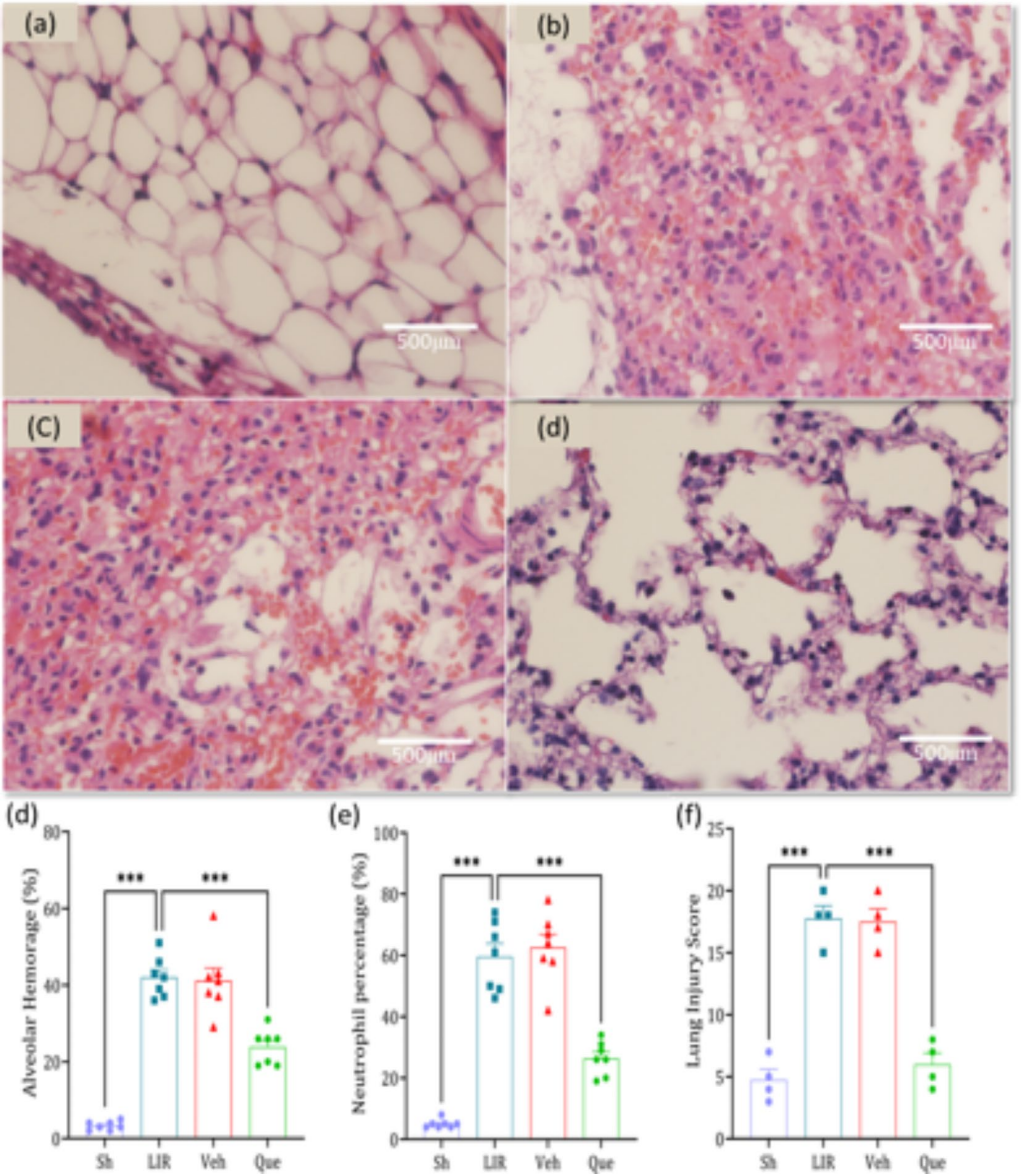
Histopathological images depicting the effects of intraperitoneal administration of quercetin on histopathological indices of lung tissue(*n* = 6). Magnification 10X. * Sh* sham group,* LIR* lung ischemia-reperfusion group,* Veh* vehicle group,* Que* quercetin group. *** *P* < 0.001.

Three criteria were assessed to examine the lung histopathological damage: alveolar hemorrhage, neutrophil infiltration, and lung injury score. All of these factors significantly increased in the LIR group, a stark contrast to the control group. A week before treatment with quercetin, a significant reduction in these indicators was observed compared to the group (Fig. 4. panels a–f).

## Discussion

Reestablishing blood flow and oxygen after ischemia provides a suitable platform for generating free radicals and inflammation, known as Lung Ischemia-Reperfusion Injury^5^. It has been demonstrated that the Nrf2/Keap1/HO-1 pathway is a crucial cellular mechanism that safeguards cells against oxidative stress, apoptosis, and inflammation^7^. Nrf2 is a significant factor in the expression of anti-inflammatory, antioxidant, and survival-supporting genes, ultimately producing proteins like HO-1, which can counteract the effects of MDA and TOS^7^.

In the present study, the effect of Quercetin on oxidative stress and inflammatory factors, lung edema index, and lung histopathology was examined in an animal model of ischemia-reperfusion lung injury. The findings indicated that ischemia-perfusion damage to the lung resulted in increased inflammation, oxidative stress, apoptosis, and lung edema, aligning with other related research.

The results of this study demonstrated the potential role of Quercetin in regulating inflammatory factors and oxidative agents, leading to a reduction in oxidative stress indicators such as MDA and TOS while increasing the levels of antioxidant factors, including Gpx, TAC, and SOD. These outcomes are consistent with the investigations conducted by Bagheri et al. in 2023 on renal tissue^22^. Furthermore, research by Zhang et al. in 2020 revealed that Quercetin possesses anti-inflammatory and antioxidant effects, preventing damage caused by myocardial ischemia reperfusion^23^.

Another study reported Quercetin as a potent antioxidant and inflammation inhibitor. Furthermore, a study by Kalich and colleagues in 2022 demonstrated that injecting Quercetin before inducing ischemia reduced lipid peroxidation, oxidative stress, and IR-induced damage in lung histopathology^24^.

Additionally, this study showed that Quercetin decreased the lung edema index. Ankit Tripathi and colleagues’ study also indicated that Quercetin could reduce the likelihood of pulmonary edema resulting from hypobaric hypoxia in rats^25^.

The present study’s results also demonstrated that Quercetin decreased the levels of inflammatory mediators such as IL-1β and TNF-α. Similar findings have been observed in other studies. For example, Mohammadrezaei and colleagues’ study in 2020 on the gastrocnemius muscle showed that Quercetin significantly reduced TNF-α and NF-κB levels^26^.

In a study conducted by Guazelli and colleagues on mice with rheumatoid arthritis inflammation, the effects of Quercetin on inflammatory parameters and immune system function were examined^27^. This study showed that Quercetin significantly reduced inflammatory parameters such as IL-1β and TNF-α and inhibited leukocyte production. This study also suggested that Quercetin’s positive effects might be related to inhibiting the NF-κB factor in the inflammatory pathway.

Furthermore, in this study, the effects of Quercetin on the expression of apoptosis-related genes were also investigated. The results demonstrated that Quercetin reduced the levels of pro-apoptotic protein Bax and, in other words, diminished the apoptosis process in cells. Moreover, Quercetin increased the expression of the anti-apoptotic protein Bcl2, thereby controlling and reducing the apoptosis process. In Ji Lu and colleagues’ study in 2021 on heart tissue^28^, as well as in You Wang and colleagues’ study in 2020 on brain tissue^15^, the use of Quercetin led to an increase in the anti-apoptotic protein Bcl2 and a decrease in the pro-apoptotic protein Bax.

Furthermore, this study demonstrated that following ischemia-reperfusion lung injury, the levels of HO-1 and Nrf2 decreased, and the level of Keap1 increased. Using Quercetin improved this pathway’s function and increased Nrf2 expression. The results of Gu and colleagues’ study in 2021 on neurons also showed an increase in Nrf2 expression after Quercetin injection^29^. In another study by Li X and colleagues, Quercetin significantly increased Nrf2 expression and improved mitochondrial function in Traumatic brain injury models^28^. Improvement in the antioxidant inhibition pathway and Nrf2 regulation was also observed in these studies.

Modulating the Nrf2/Keap1 pathway and inhibiting the NF-κB pathway by Quercetin is very important. The Nrf2/Keap1 pathway plays an essential role in defending cells against oxidative damage by regulating the expression of various antioxidant proteins. By increasing the activity of Nrf2, Quercetin promotes the transcription of genes involved in antioxidant responses, thereby reducing oxidative stress and associated cell damage. This modulation is crucial to reduce the deleterious effects of reperfusion injury, as it helps maintain cellular homeostasis and prevents excessive inflammatory responses.

On the other hand, inhibition of NF-κB pathway by Quercetin emphasizes its anti-inflammatory potential. NF-κB is a crucial regulator of inflammation, and its activation leads to the expression of pro-inflammatory cytokines such as TNF-α and IL-1β. Therefore, by inhibiting the activity of NF-κB by Quercetin, the production of these cytokines decreases, reducing inflammation and its harmful effects on tissues. This anti-inflammatory effect complements the antioxidant effects of Quercetin and provides a comprehensive protective mechanism against ischemia-reperfusion injury.

By integrating these mechanisms, Quercetin provides a multifaceted approach to cellular protection crucial for developing therapeutic strategies against ischemia-reperfusion injury.

## Conclusion

In this study, we arrived at findings that indicate the protective effects of Quercetin against lung ischemia-reperfusion. Furthermore, it was observed that quercetin administration leads to the activation of the Nrf2/Keap1/HO-1 pathway, reducing inflammatory and oxidative factors. Quercetin’s effects in preventing the decline of antioxidant mediators’ activity were also obtained. Further research is needed to uncover the precise mechanisms of Quercetin’s effects.

## Supplementary Information

Supplementary Information

## Data Availability

The datasets analyzed during the current study are not publicly available but are available from the corresponding author on reasonable request.

## Abbreviations

LIRI: Lung ischemia-reperfusion injury
Nrf2: Nuclear factor erythroid 2-related factor 2
Keap1: Kelch-like ECH-associated protein 1
HO-1: Heme oxygenase 1
Bax: Bcl-2–associated X protein
Bcl2: B-cell leukemia/lymphoma 2 protein
NF-κB: Nuclear factor kappa-light-chain-enhancer of activated B cells
PGD: Primary graft dysfunction
OBS: Obstructive bronchiolitis syndrome
N_2_O: Nitrous oxide
IR: Ischemia reperfusion
BALF: Bronchoalveolar lavage fluid
ELISA: Enzyme-linked Immunosorbent assay
SOD: Superoxide dismutase
Gpx: Glutathione peroxidase
MDA: Malondialdehyde
TOS: Total oxidant status
TAC: Total antioxidant capacity
TNF-α: Tumor necrosis factor alpha
IL-1β: Interleukin-1β
ECL: Enhanced chemiluminescence
