# Supplementary material for: Quercetin as a therapeutic agent activate the Nrf2/Keap1 pathway to alleviate lung ischemia-reperfusion injury
Source: Sci Rep. 2024 Oct 4;14:23074. doi: 10.1038/s41598-024-73075-7 (PMC11452703; doi:10.1038/s41598-024-73075-7)
Supplement: Supplementary file 1 — Supplementary Information [file 41598_2024_73075_MOESM1_ESM.pdf]

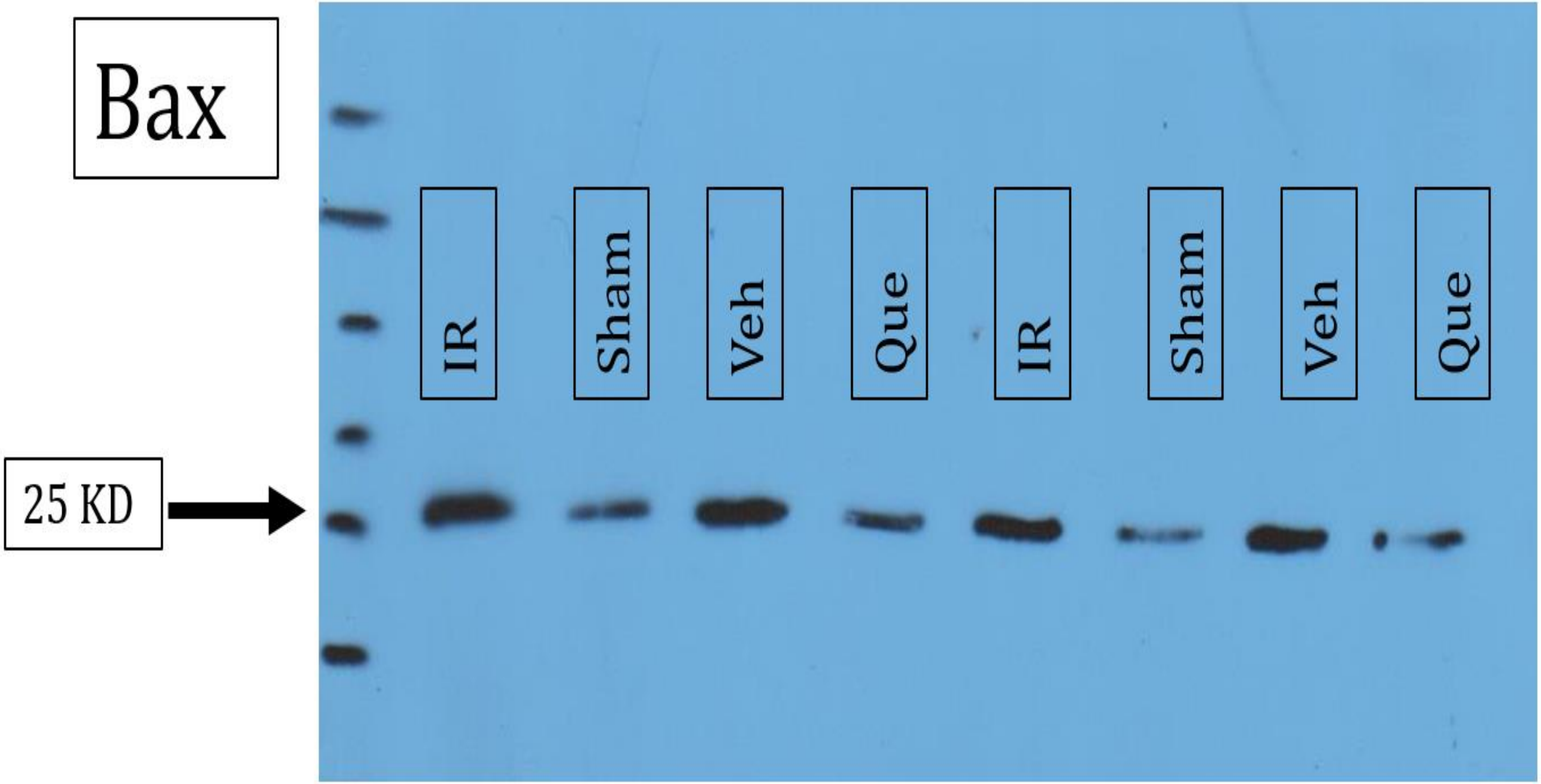
Bax
25 KD
IR
Sham
Veh
Que
IR
Sham
Veh
Que

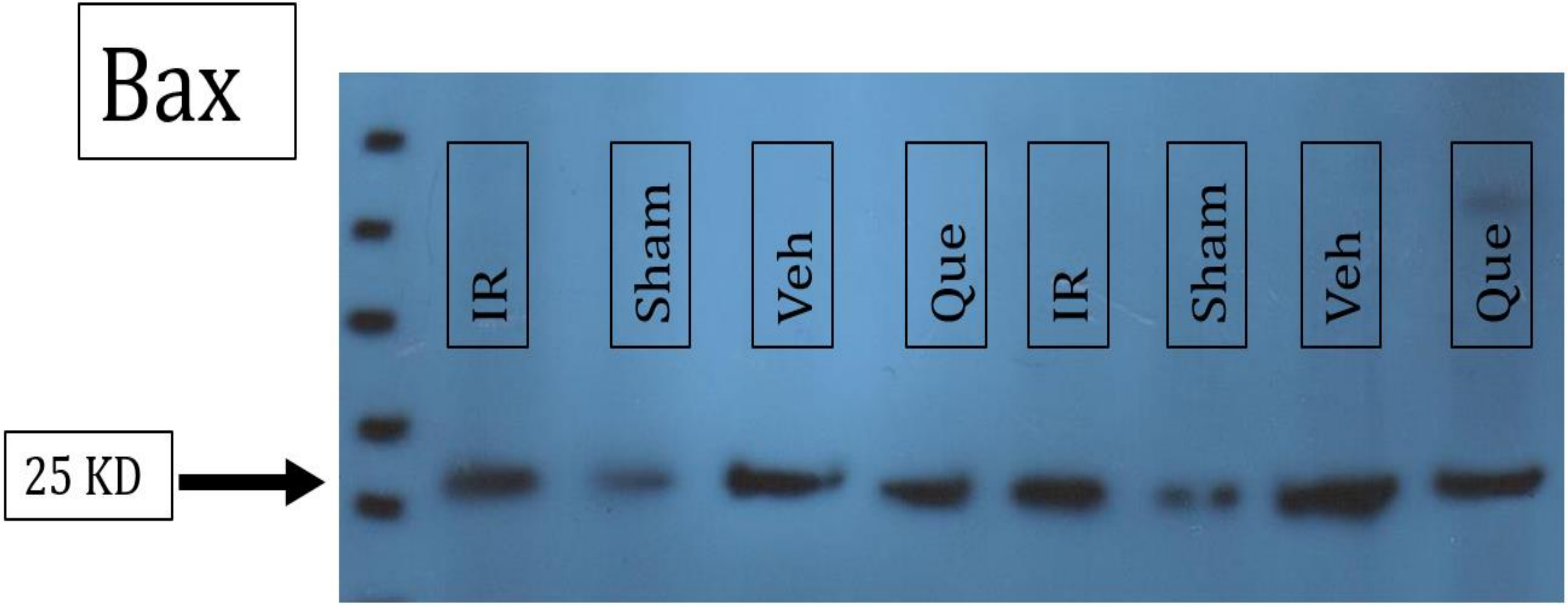
Bax
25 KD
IR
Sham
Veh
Que
IR
Sham
Veh
Que

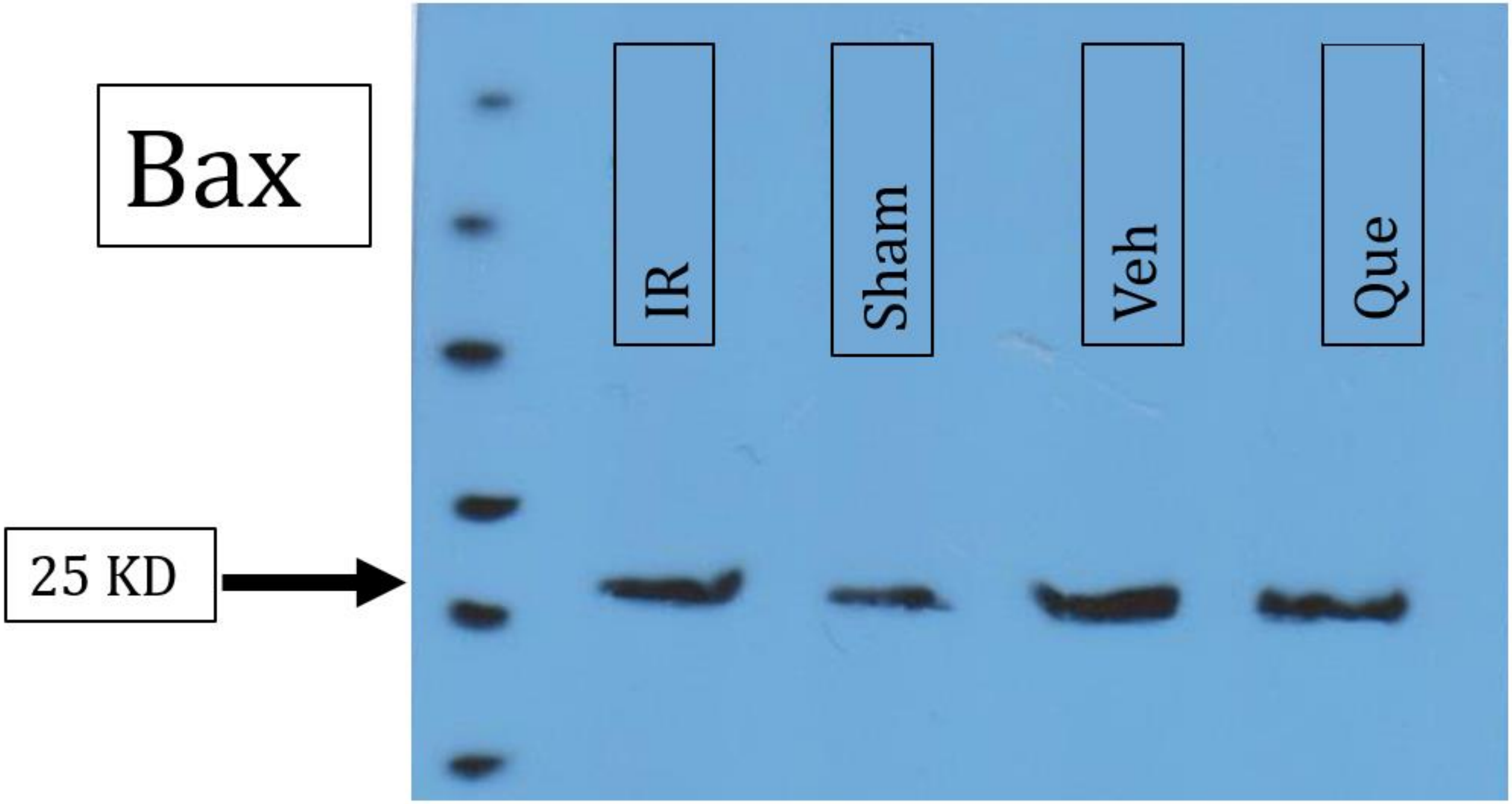
Bax
25 KD
IR
Sham
Veh
Que

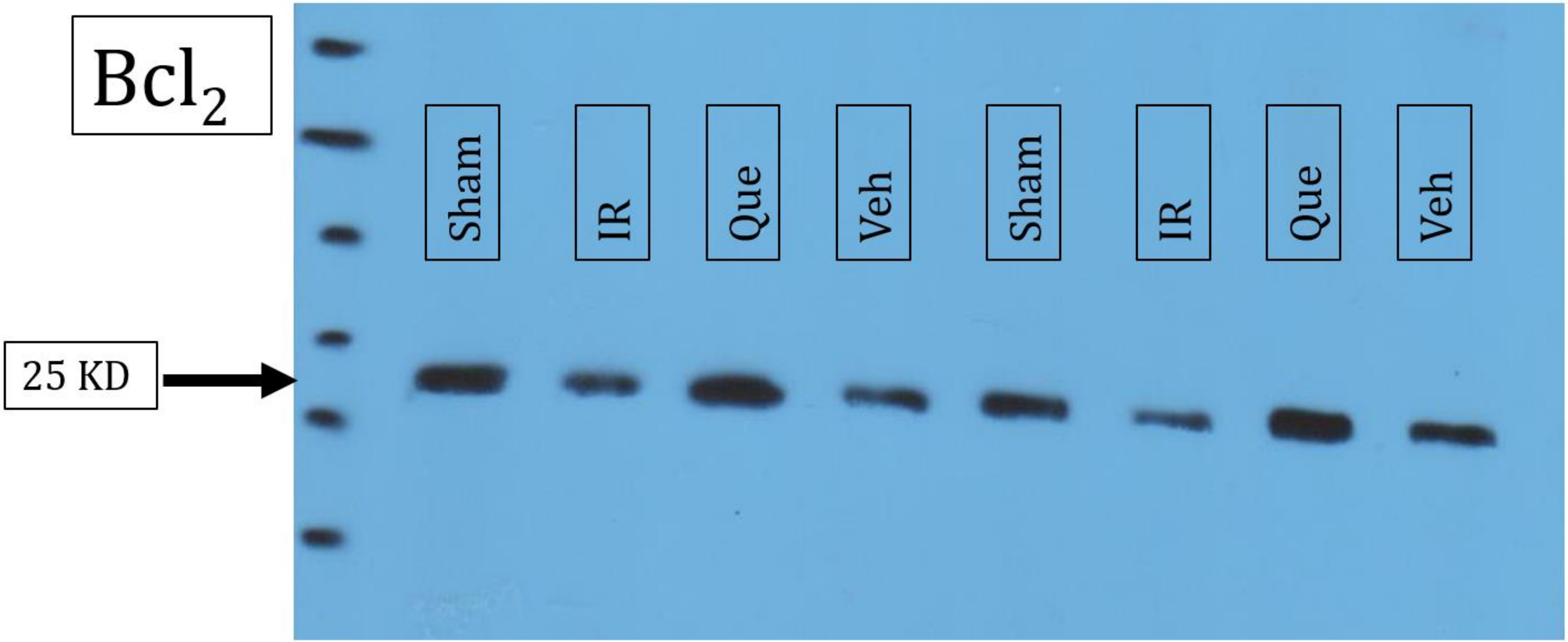
Bcl2
25 KD
Sham
IR
Que
Veh
Sham
IR
Que
Veh

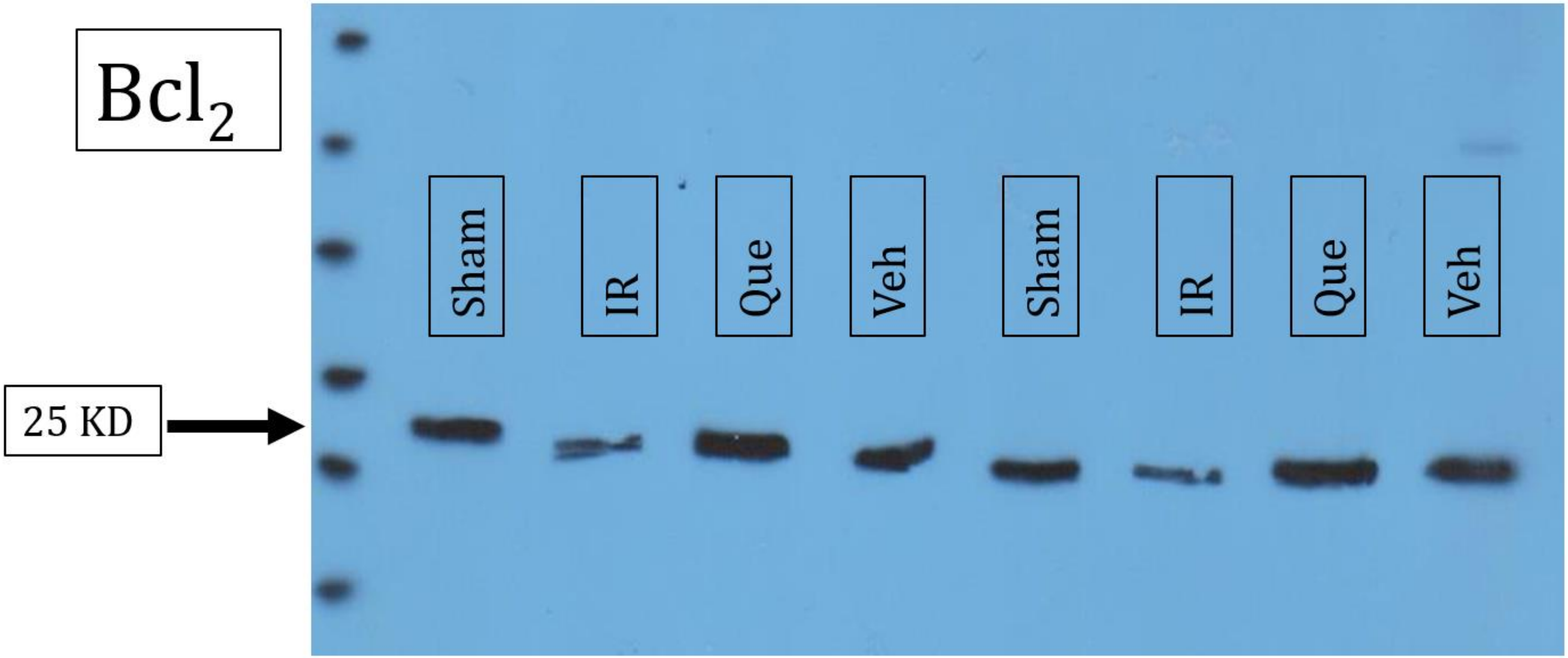

Bcl$_2$
25 KD
Sham
IR
Que
Veh
Sham
IR
Que
Veh

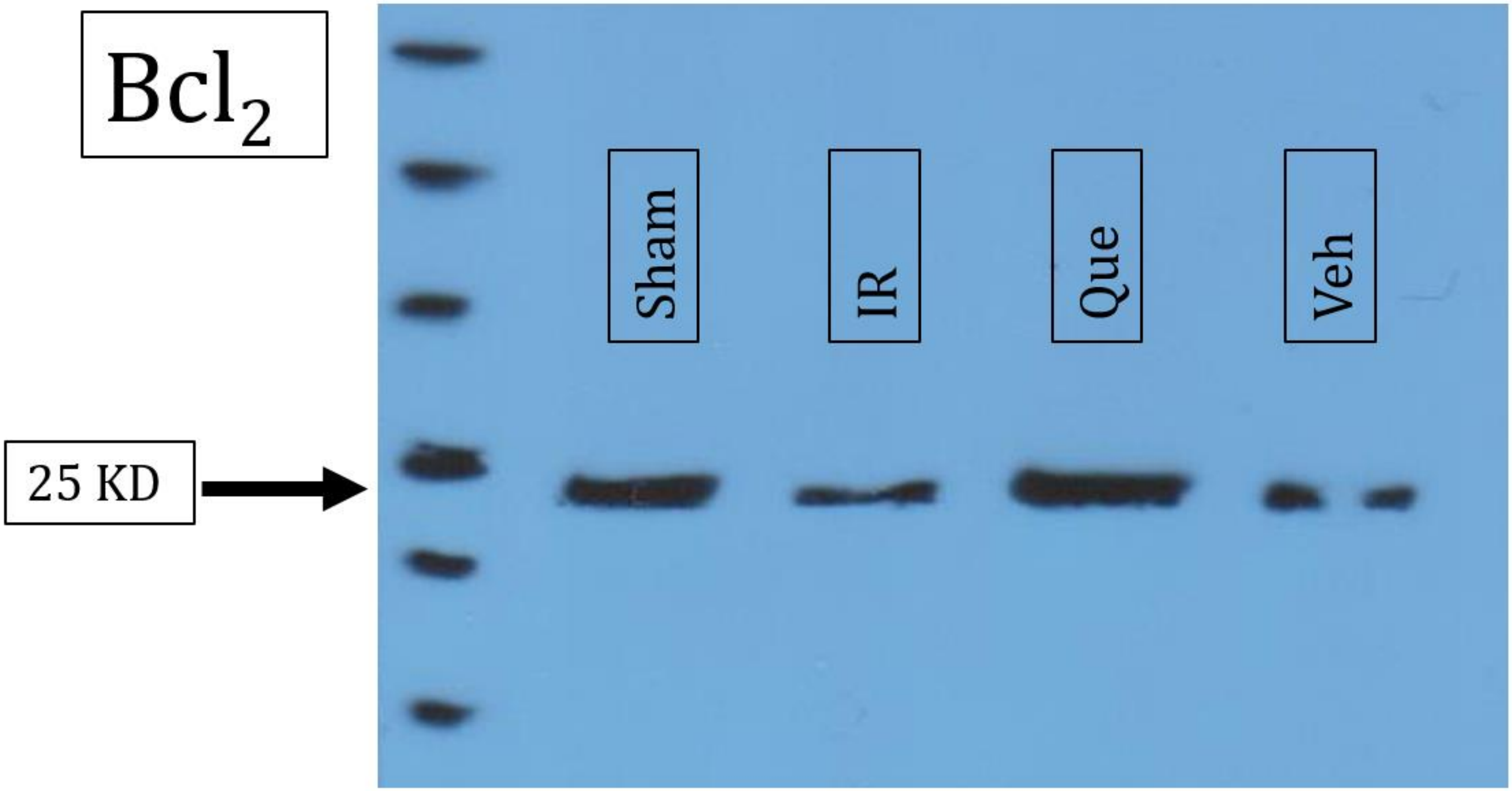

Bcl_2
25 KD
Sham
IR
Que
Veh

HO-1

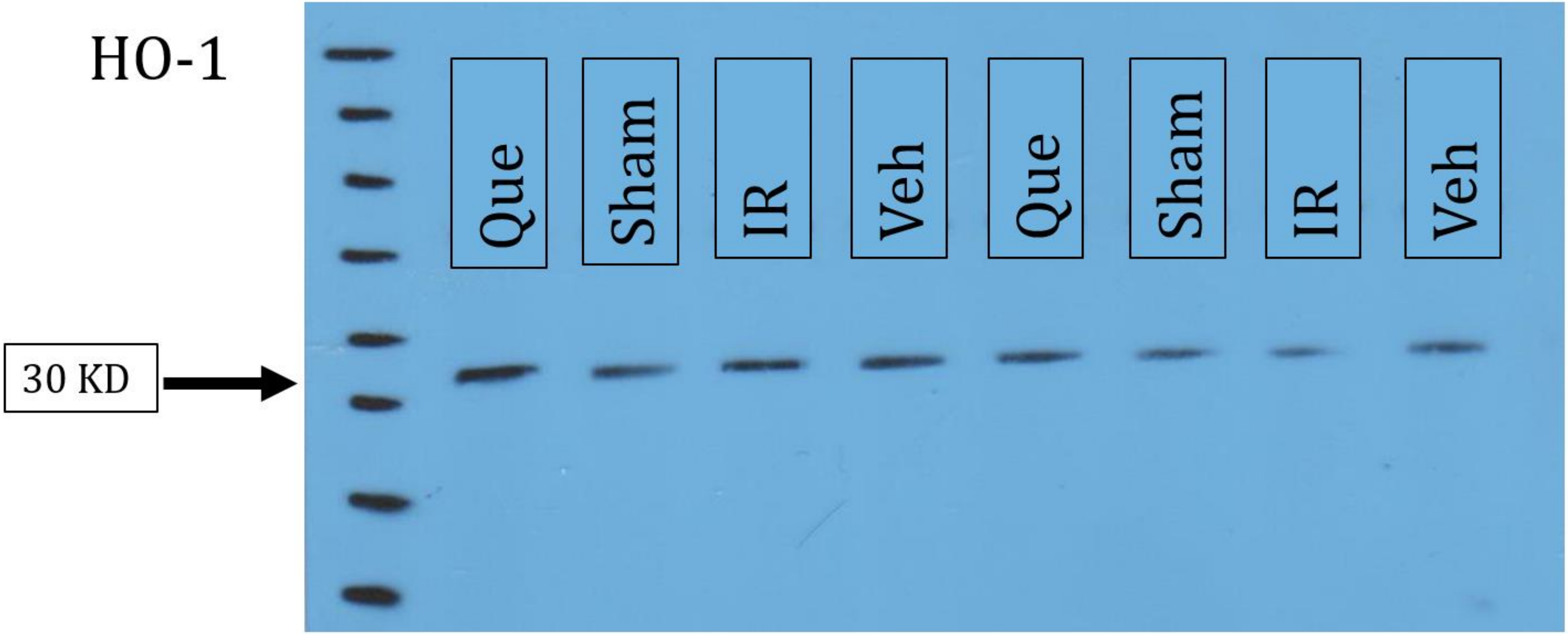

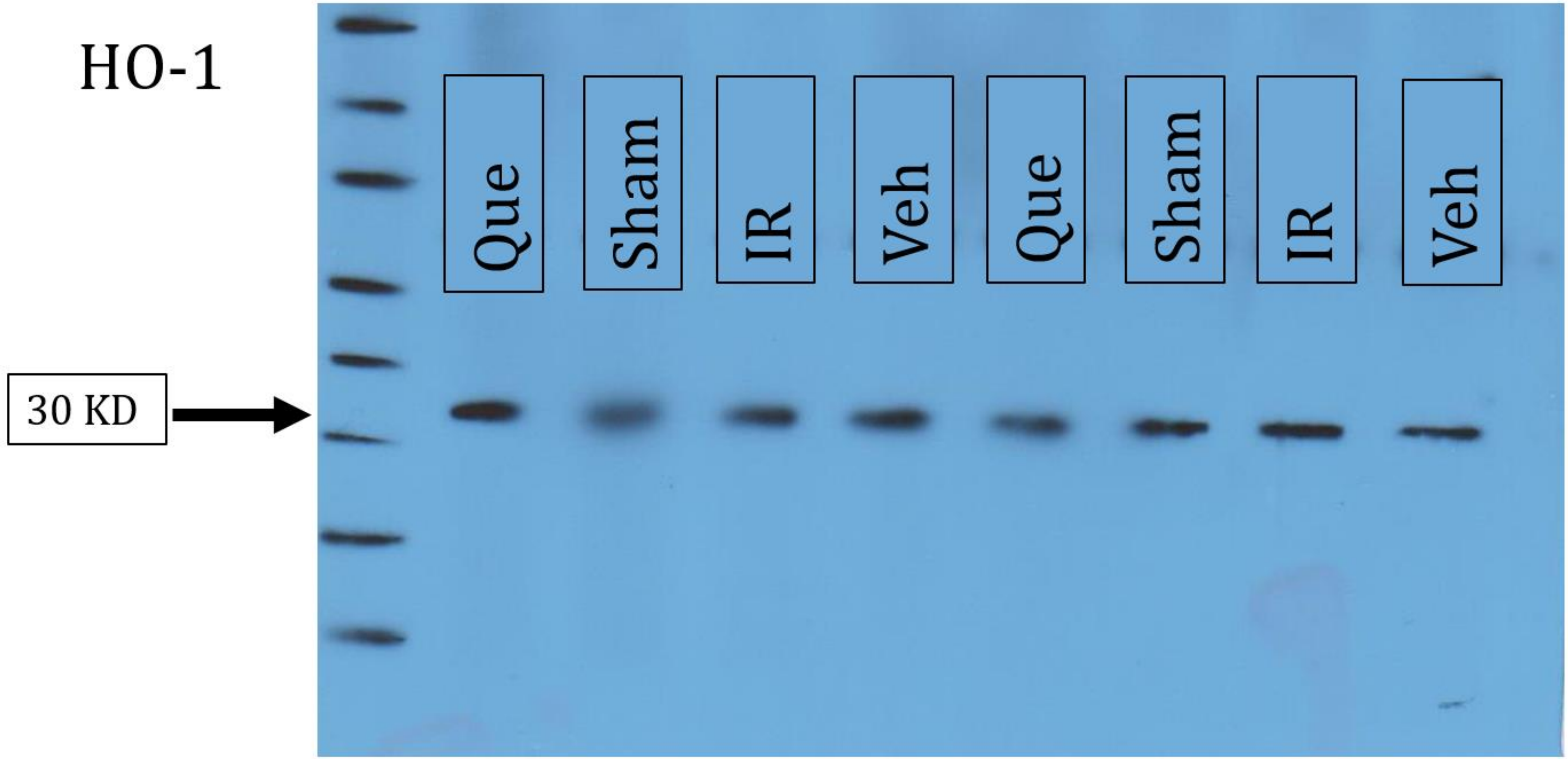
HO-1
30 KD
Que
Sham
IR
Veh
Que
Sham
IR
Veh

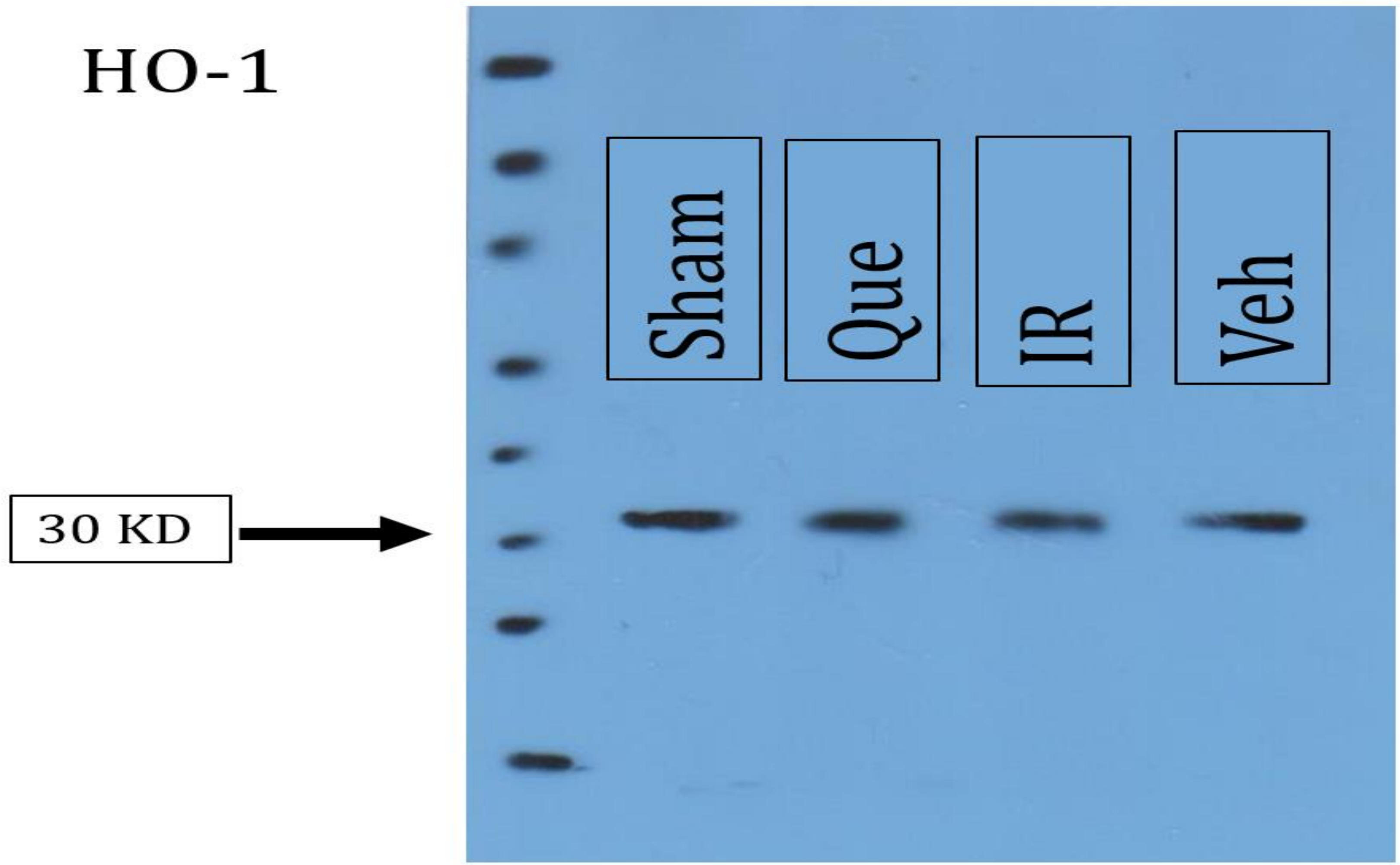

HO-1
Sham
Que
IR
Veh
30 KD

Keap-1

65 KD

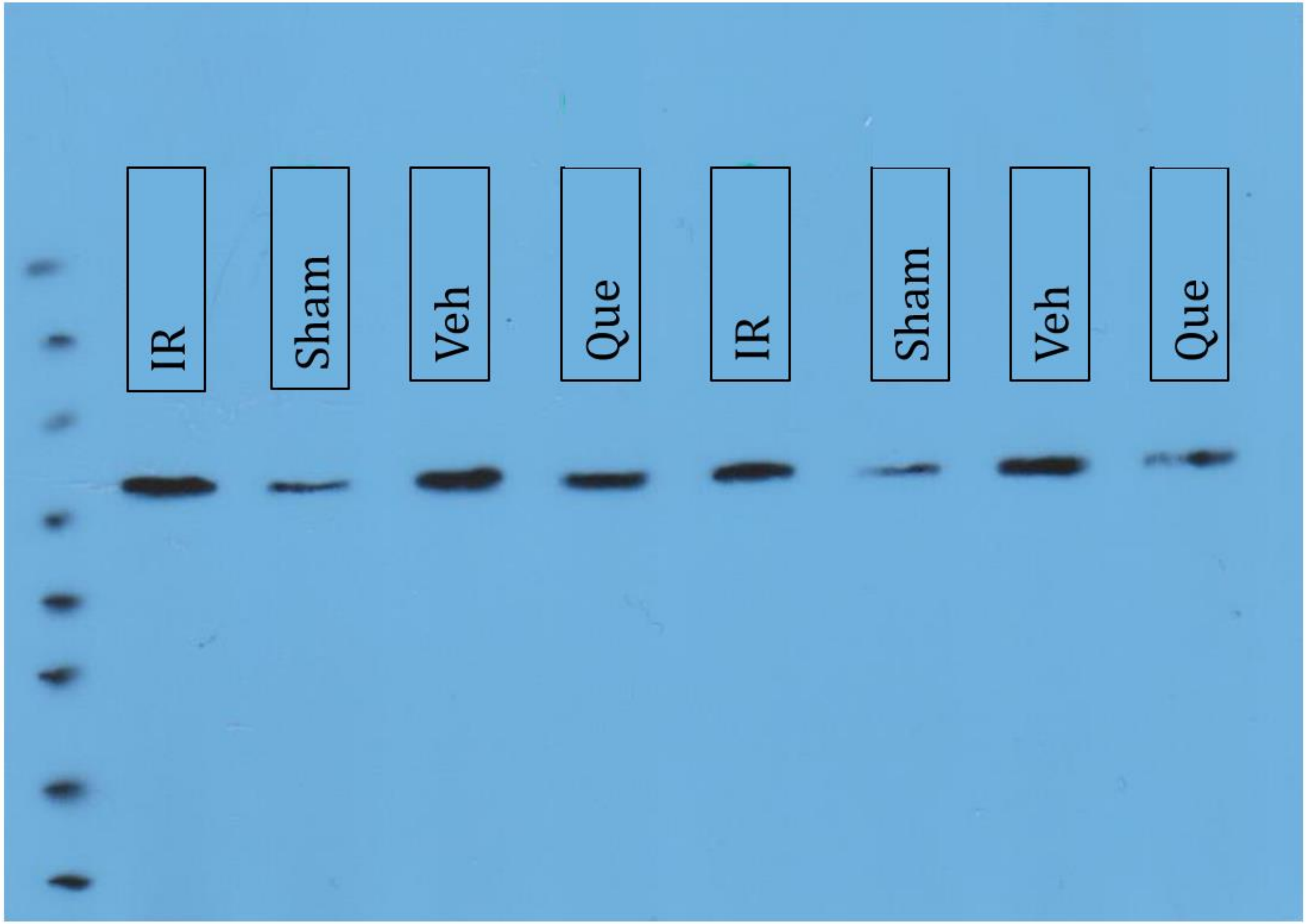

Keap-1

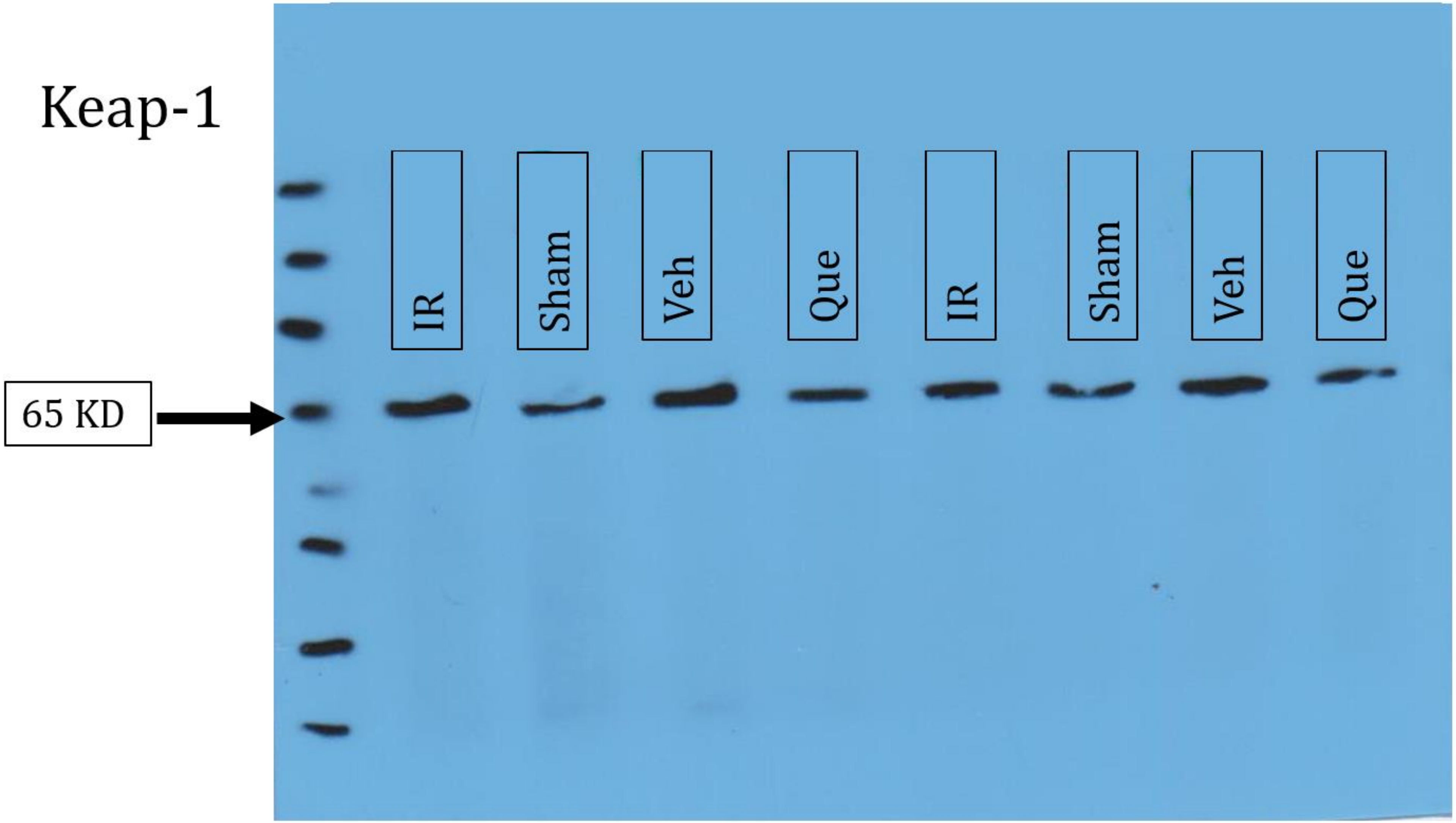

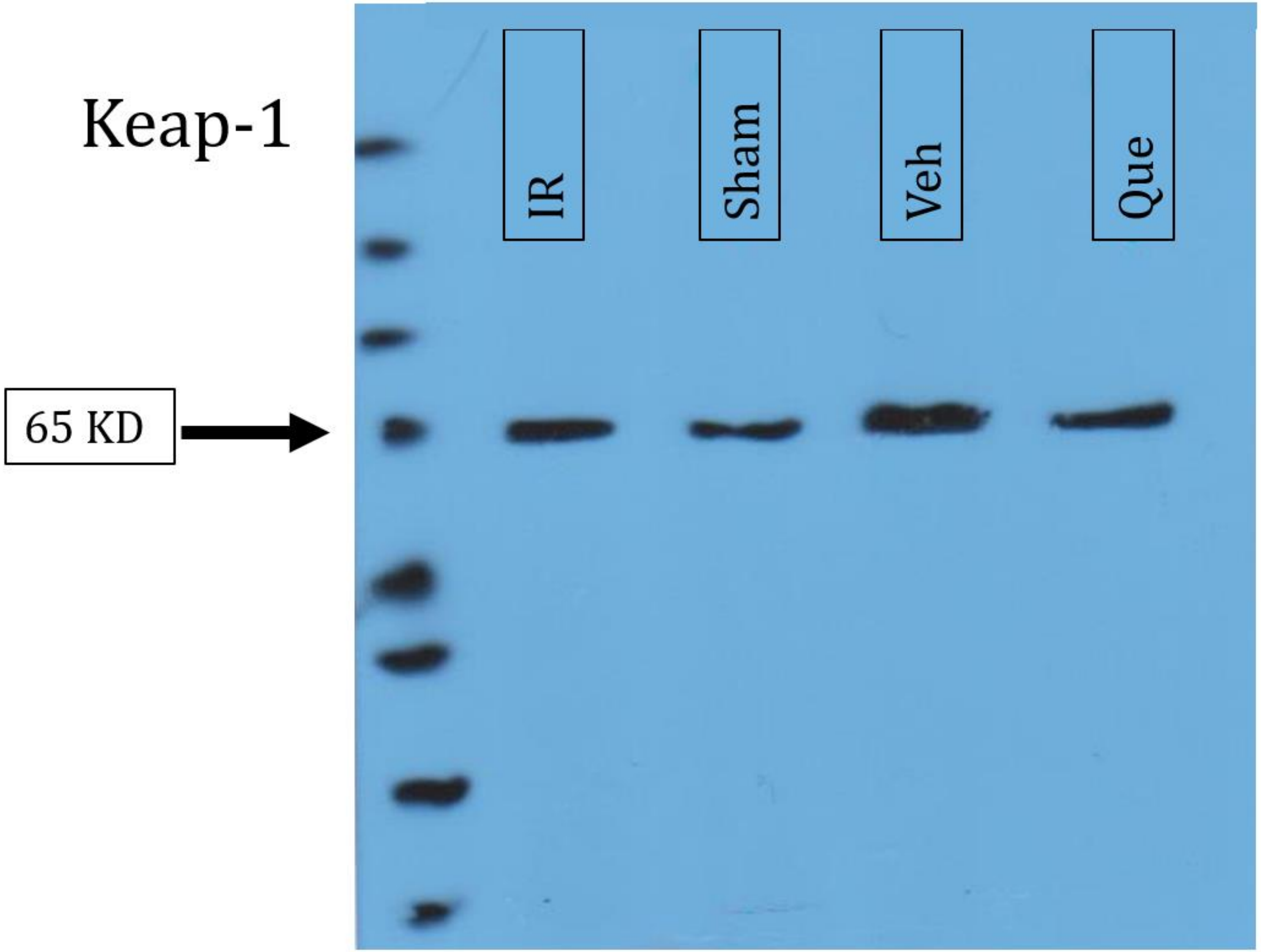
Keap-1
65 KD
IR
Sham
Veh
Que

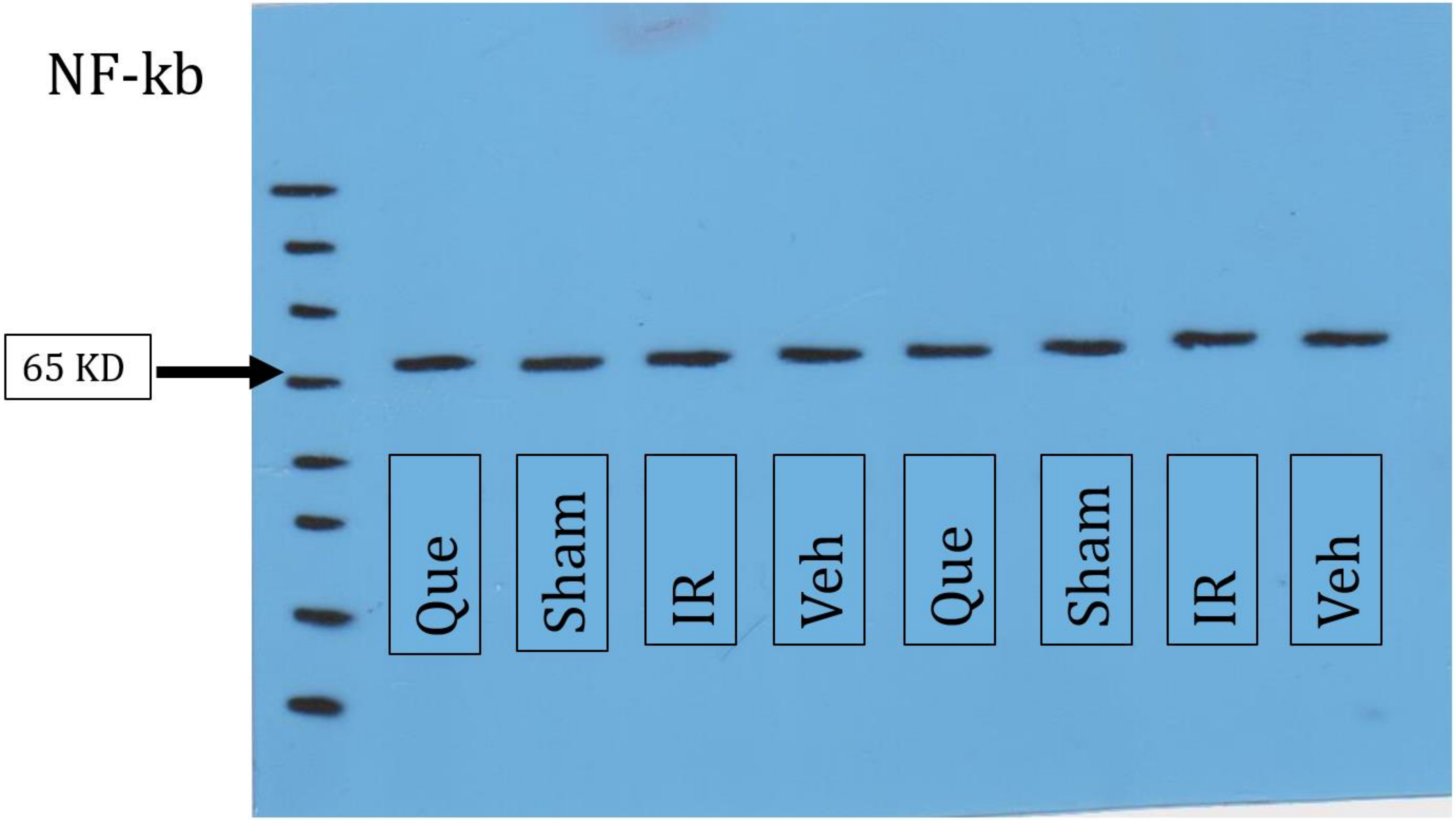
NF-kb
65 KD
Que
Sham
IR
Veh
Que
Sham
IR
Veh

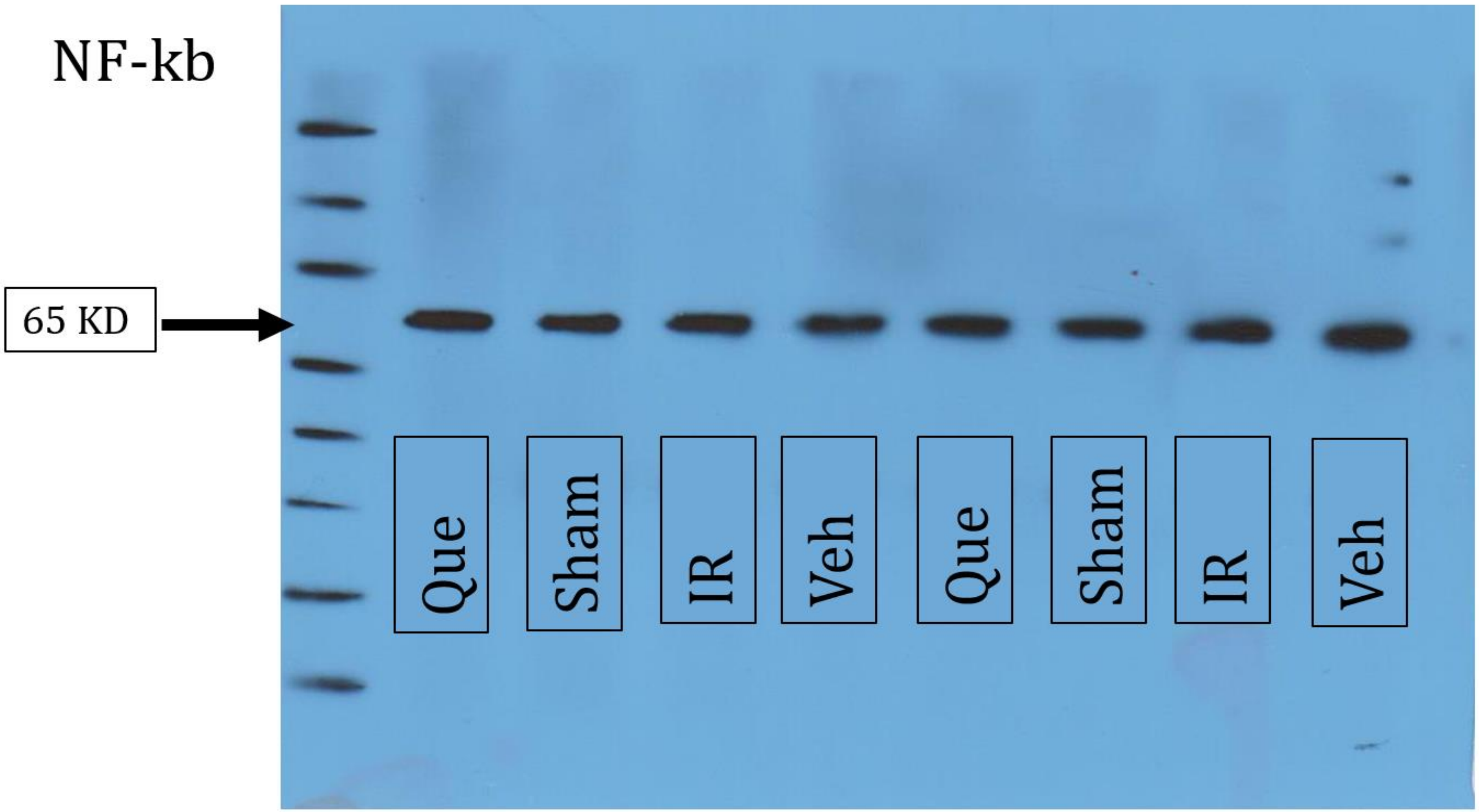
NF-kb
65 KD
Que
Sham
IR
Veh
Que
Sham
IR
Veh

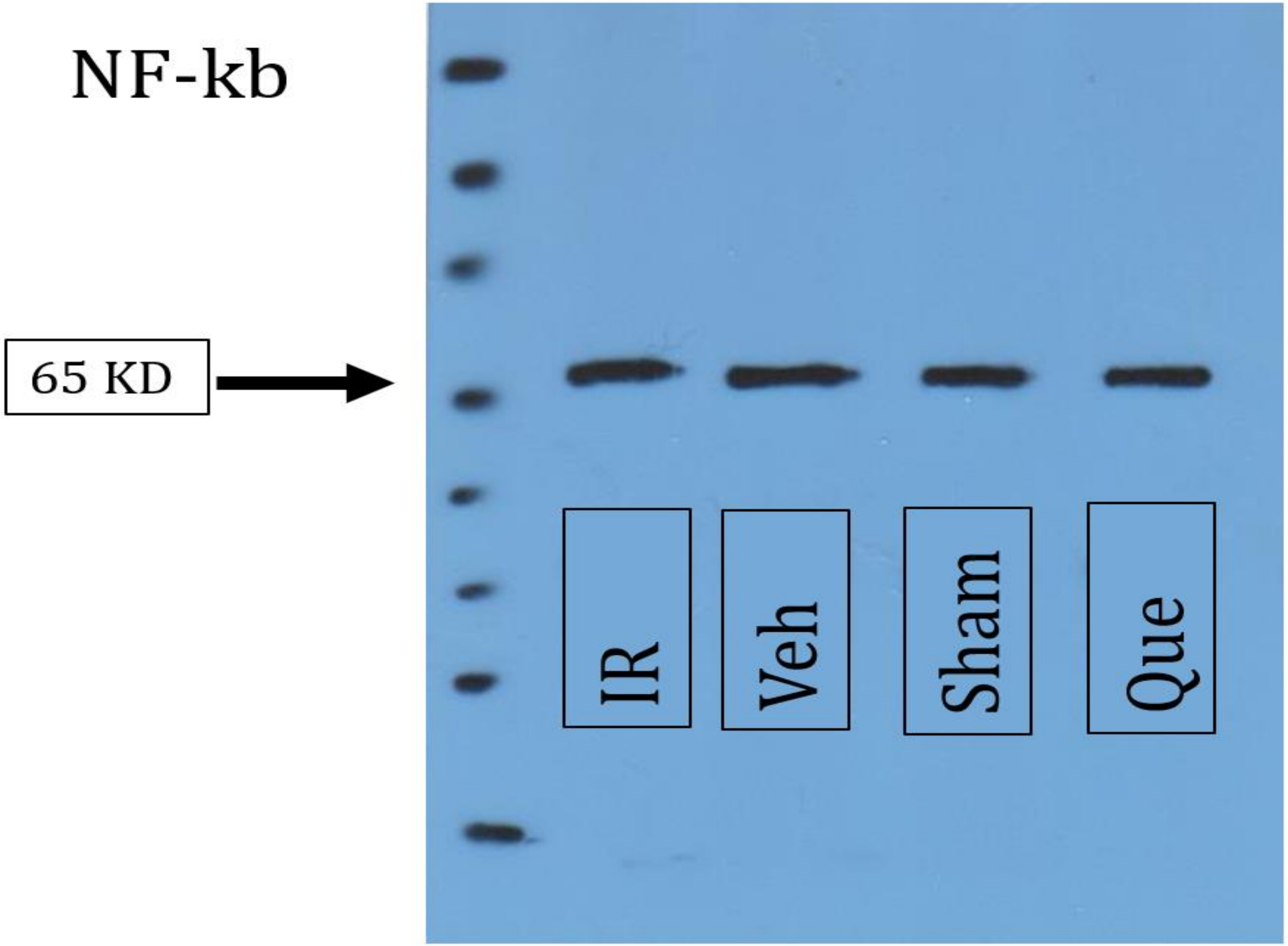
NF-kb
65 KD
IR
Veh
Sham
Que

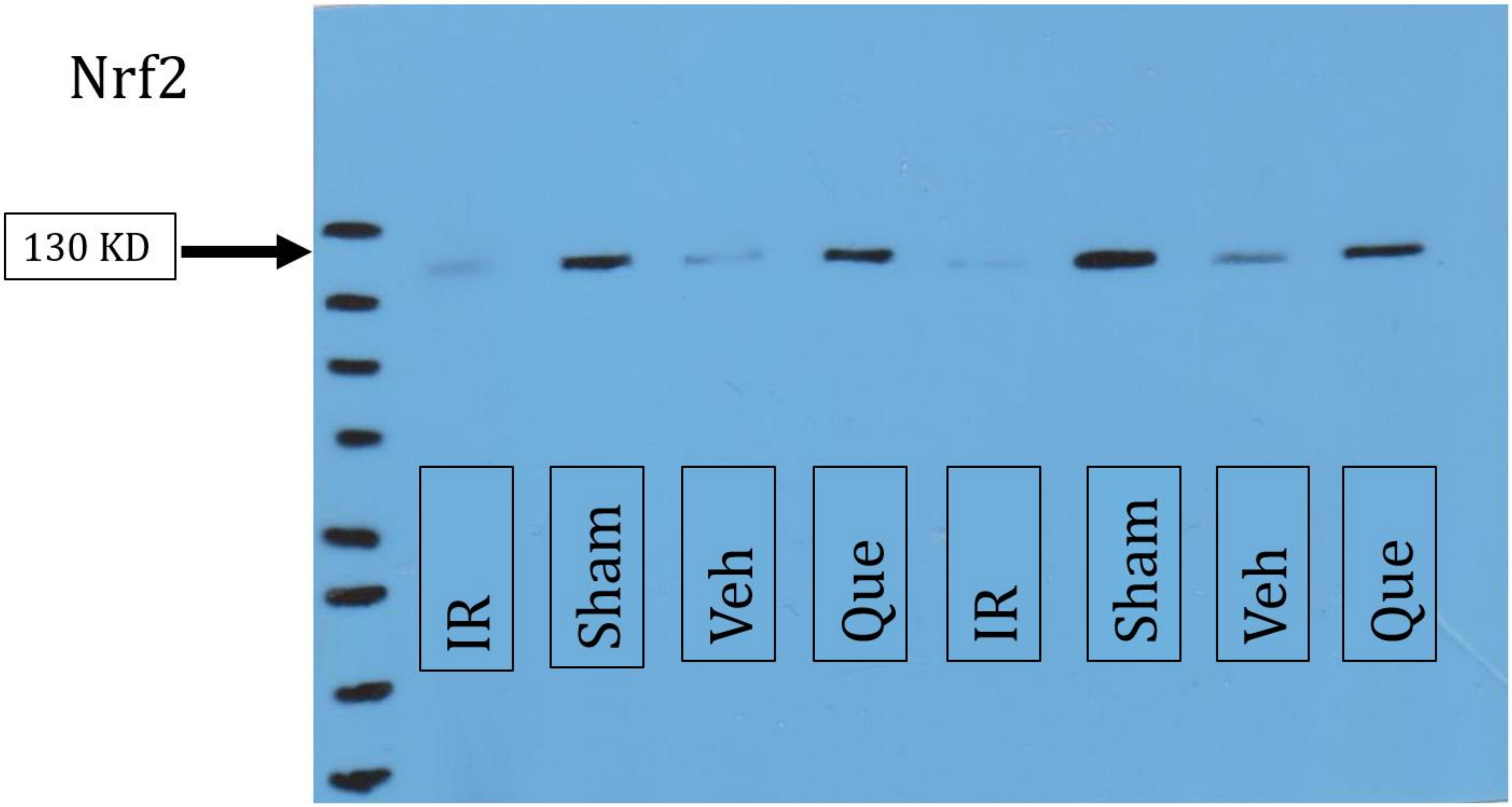
Nrf2
130 KD
IR
Sham
Veh
Que
IR
Sham
Veh
Que

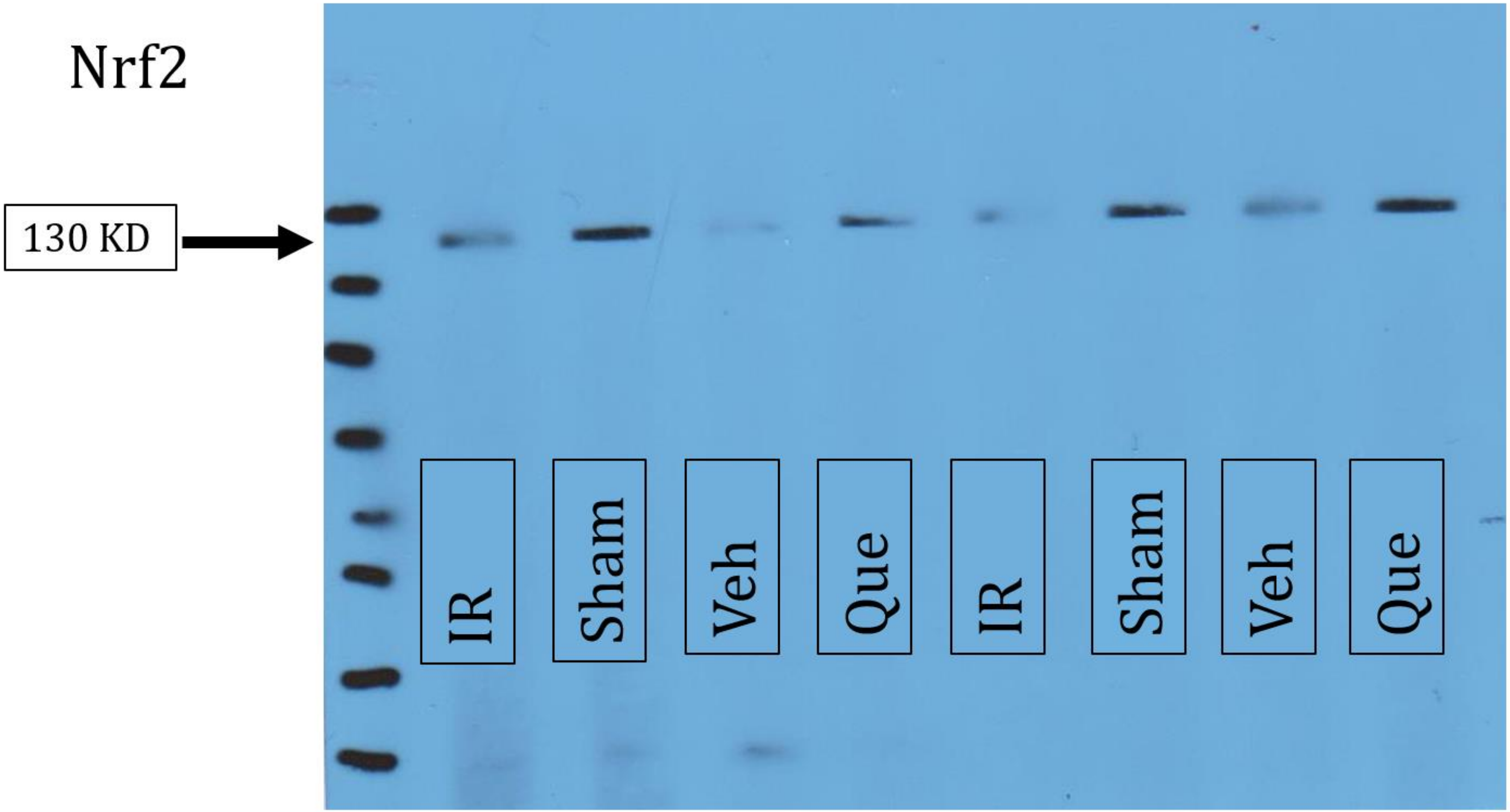
Nrf2
130 KD
IR
Sham
Veh
Que
IR
Sham
Veh
Que

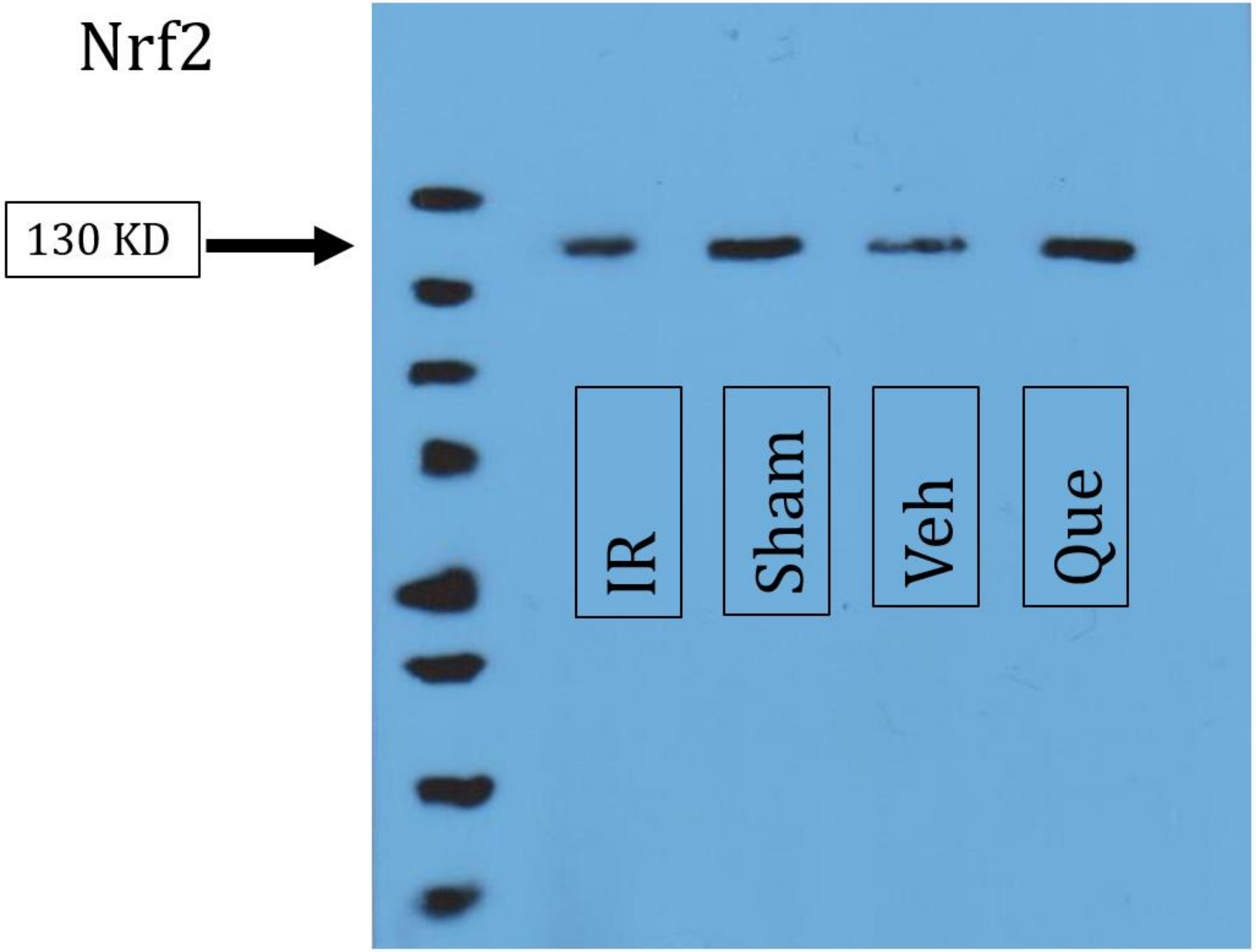

Nrf2
130 KD
IR
Sham
Veh
Que

Beta-Actin

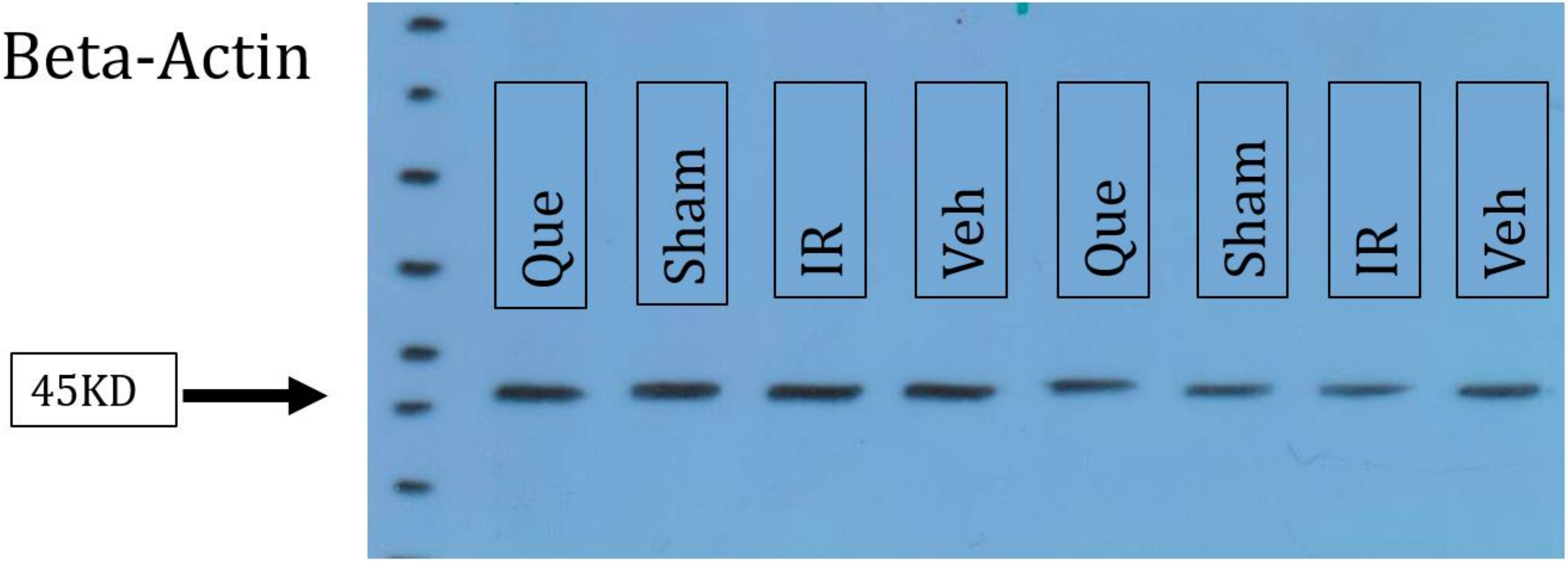

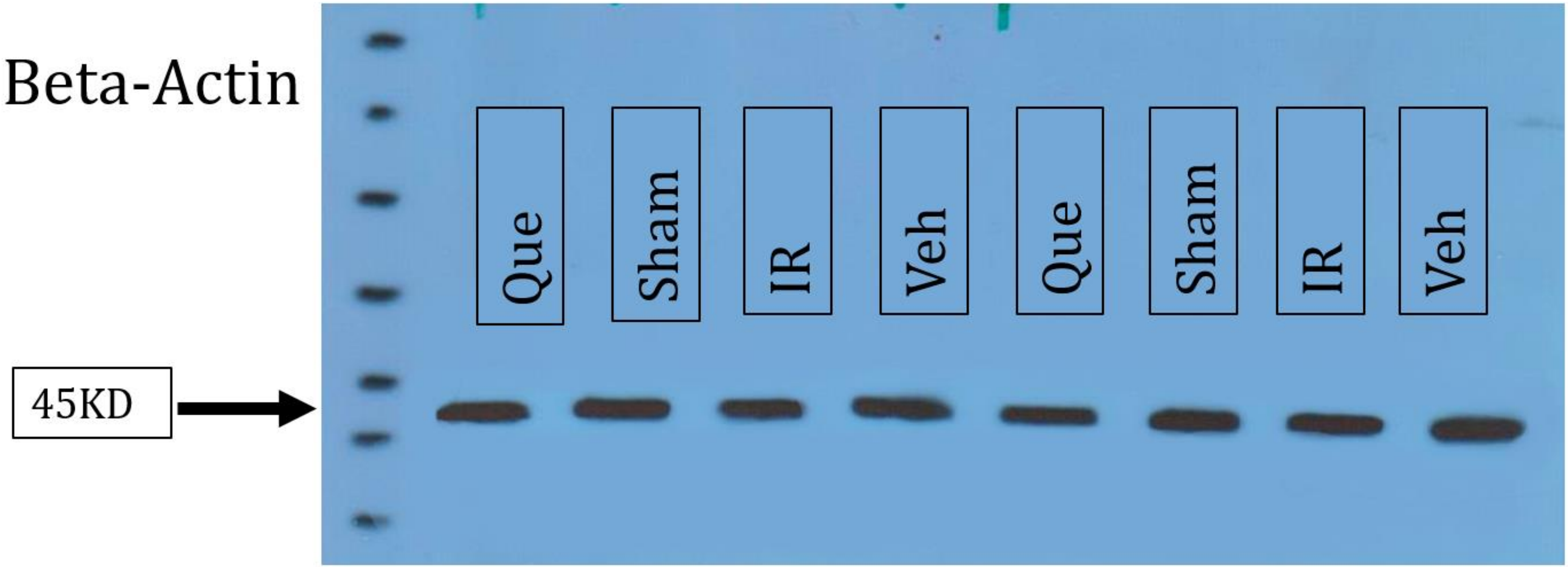
Beta-Actin
Que
Sham
IR
Veh
Que
Sham
IR
Veh
45KD

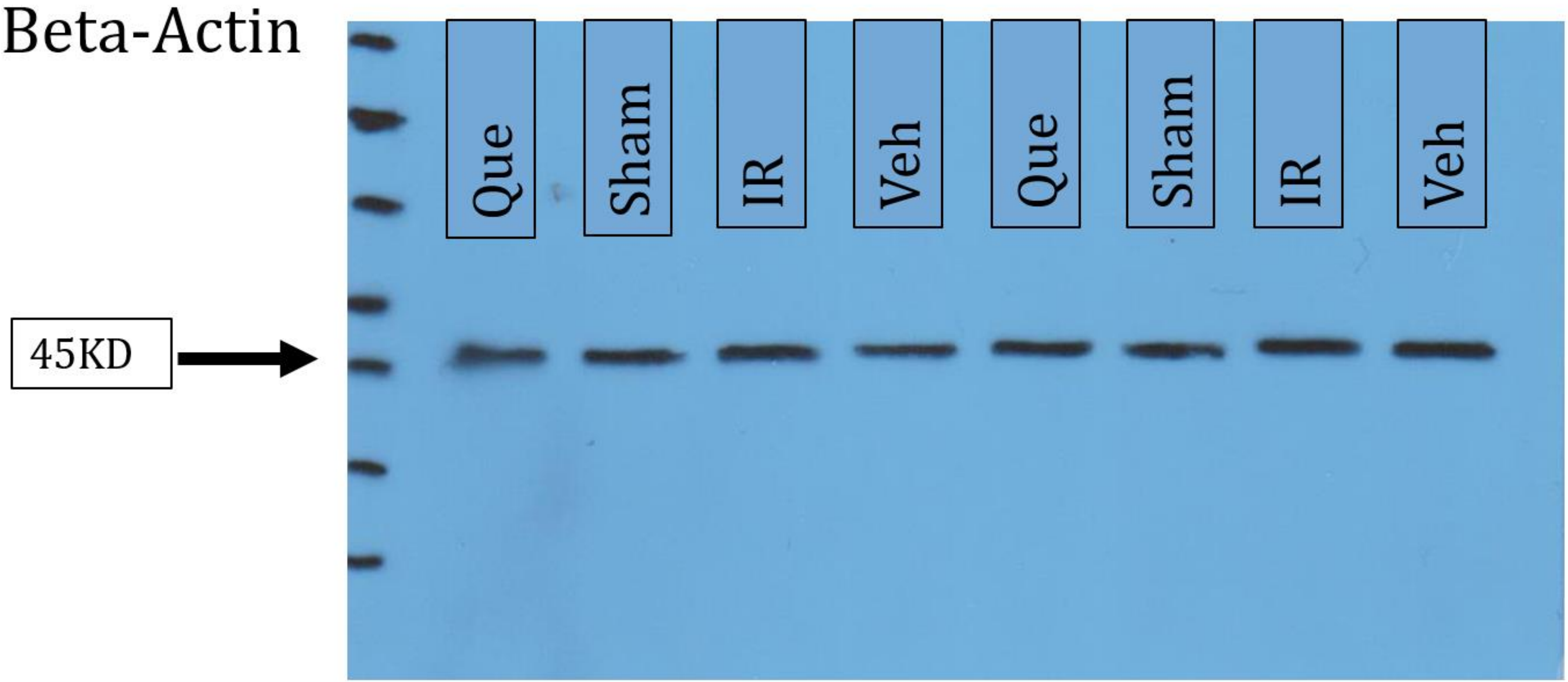
Beta-Actin
45KD
Que
Sham
IR
Veh
Que
Sham
IR
Veh

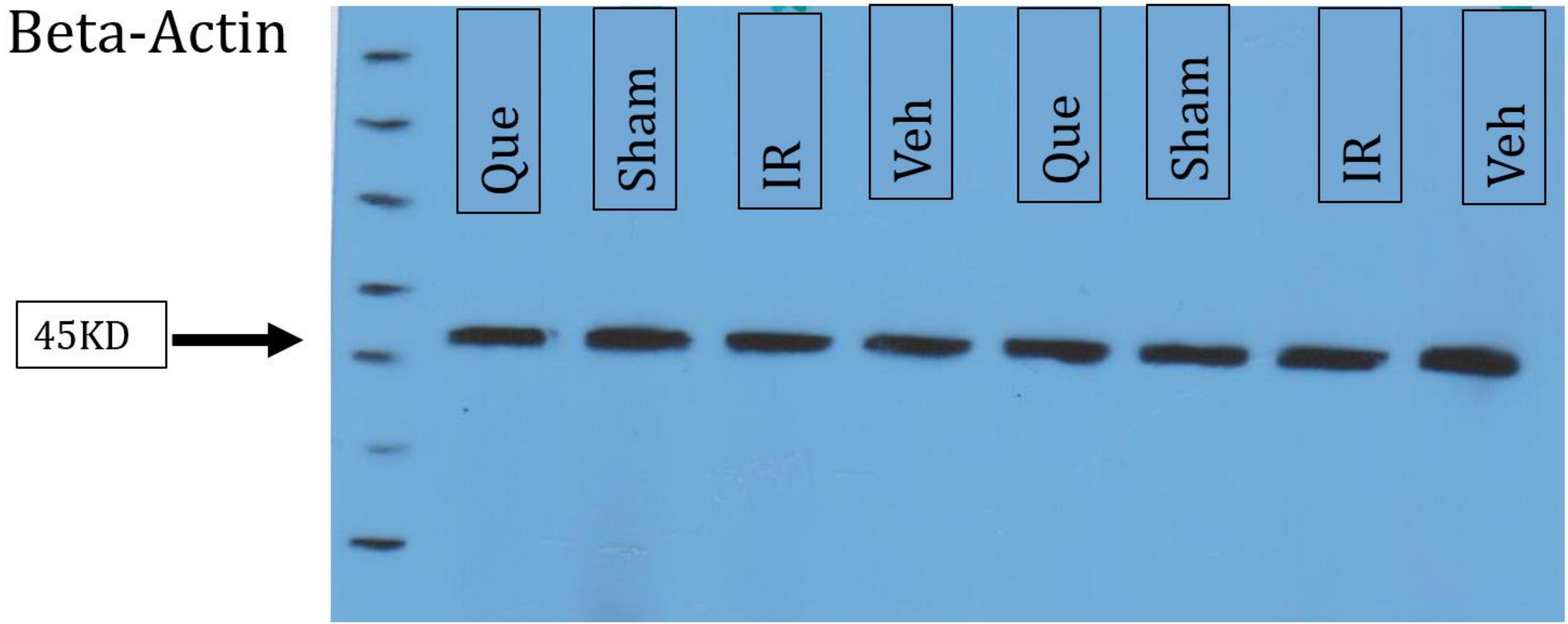

Beta-Actin
45KD
Que
Sham
IR
Veh
Que
Sham
IR
Veh
